# Ago HITS-CLIP Expands Understanding of Kaposi's Sarcoma-associated Herpesvirus miRNA Function in Primary Effusion Lymphomas

**DOI:** 10.1371/journal.ppat.1002884

**Published:** 2012-08-23

**Authors:** Irina Haecker, Lauren A. Gay, Yajie Yang, Jianhong Hu, Alison M. Morse, Lauren M. McIntyre, Rolf Renne

**Affiliations:** 1 Department of Molecular Genetics and Microbiology, University of Florida, Gainesville, Florida, United States of America; 2 UF Genetics Institute, University of Florida, Gainesville, Florida, United States of America; 3 UF Shands Cancer Center, University of Florida, Gainesville, Florida, United States of America; University of North Carolina at Chapel Hill, United States of America

## Abstract

KSHV is the etiological agent of Kaposi's sarcoma (KS), primary effusion lymphoma (PEL), and a subset of multicentricCastleman's disease (MCD). The fact that KSHV-encoded miRNAs are readily detectable in all KSHV-associated tumors suggests a potential role in viral pathogenesis and tumorigenesis. MiRNA-mediated regulation of gene expression is a complex network with each miRNA having many potential targets, and to date only few KSHV miRNA targets have been experimentally determined. A detailed understanding of KSHV miRNA functions requires high-through putribonomics to globally analyze putative miRNA targets in a cell type-specific manner. We performed Ago HITS-CLIP to identify viral and cellular miRNAs and their cognate targets in two latently KSHV-infected PEL cell lines. Ago HITS-CLIP recovered 1170 and 950 cellular KSHVmiRNA targets from BCBL-1 and BC-3, respectively. Importantly, enriched clusters contained KSHV miRNA seed matches in the 3′UTRs of numerous well characterized targets, among them *THBS1*, *BACH1*, and *C/EBPβ*. KSHV miRNA targets were strongly enriched for genes involved in multiple pathways central for KSHV biology, such as apoptosis, cell cycle regulation, lymphocyte proliferation, and immune evasion, thus further supporting a role in KSHV pathogenesis and potentially tumorigenesis. A limited number of viral transcripts were also enriched by HITS-CLIP including *vIL-6* expressed only in a subset of PEL cells during latency. Interestingly, Ago HITS-CLIP revealed extremely high levels of Ago-associated KSHV miRNAs especially in BC-3 cells where more than 70% of all miRNAs are of viral origin. This suggests that in addition to seed match-specific targeting of cellular genes, KSHV miRNAs may also function by hijacking RISCs, thereby contributing to a global de-repression of cellular gene expression due to the loss of regulation by human miRNAs. In summary, we provide an extensive list of cellular and viral miRNA targets representing an important resource to decipher KSHV miRNA function.

## Author Summary

Kaposi's sarcoma-associated herpesvirus is the etiological agent of KS and two lymphoproliferative diseases: multicentricCastleman's disease and primary effusion lymphomas (PEL). KSHV tumors are the most prevalent AIDS malignancies and within Sub-Saharan Africa KS is the most common cancer in males, both in the presence and absence of HIV infection. KSHV encodes 12 miRNA genes whose function is largely unknown. Viral miRNAs are incorporated into RISCs, which regulate gene expression mostly by binding to 3′UTRs of mRNAs to inhibit their translation and/or induce degradation. The small subset of viral miRNA targets identified to date suggests that these small posttranscriptional regulators target important cellular pathways involved in pathogenesis and tumorgenesis. Using Ago HITS-CLIP, a technique which combines UV cross-linking, immunoprecipitation of Ago-miRNA-mRNA complexes, and high throughput sequencing, we performed a detailed analysis of the KSHV miRNA targetome in two commonly studied PEL cell lines, BCBL-1 and BC-3 and identified 1170 and 950 putative miRNA targets, respectively. This data set provides a valuable resource to decipher how KSHV miRNAs contribute to viral biology and pathogenesis.

## Introduction

Kaposi's sarcoma-associated herpesvirus (KSHV) or Human Herpesvirus type 8 (HHV-8) is associated with Kaposi's sarcoma (KS) and two lymphoproliferative disorders: primary effusion lymphomas (PEL) and a subset of multicentricCastleman's disease (MCD) [1]–[3]. In KS tumors and PEL viral gene expression is highly restricted to the latency-associated region which encodes four proteins and the viral microRNAs(miRNA). MiRNAs are 21 to 23 nucleotide (nt) long, non-coding RNAs that preferentially bind to 3′UTRs of mRNAs to prevent translation and/or induce degradation (for review see [4]). The first viral miRNAs were identified in 2004 in Epstein-Barr virus (EBV)-infected Burkitt's lymphoma cells [5] and subsequently more than 140 miRNAs have been identified in all herpes viruses studied thus far with the exception of Varicella Zoster virus (for review see [6], [7]). The 12 KSHV miRNA genes [8]–[11] can each give rise to two different mature products [12], miR and miR*. MiR-K12-10 is moreover edited [13] bringing the total number of mature miRNAs to 25. KSHV miRNAs are expressed during the latent phase of infection and expression has been detected in tissues and biopsies of classical and AIDS-associated KS as well as in PEL and MCD [14]–[16]. Since aberrant expression of miRNAs is associated with many human diseases including cancer [17], it was hypothesized early on that KSHV-encoded miRNAs may contribute to pathogenesis and/or tumorigenesis by de-regulating host cellular gene expression. Until recently, only a small number of target genes have been identified mainly by combining bioinformatics predictions with gene expression profiling and 3′UTR luciferase reporter assays in cells that either ectopically express viral miRNAs or in tumor cell lines in which viral miRNAs are inhibited by antagomir approaches [18]–[21]. Although limited in number, the initially reported targets immediately suggested that KSHVmiRNAs contribute to the regulation of pathogenesis-relevant processes such as angiogenesis, apoptosis, cell cycle control, endothelial cell differentiation, and immune surveillance (for review see [6], [7]). Moreover, one KSHV miRNA, miR-K12-11, shares the same seed sequence as human miR-155, one of the first “oncomirs” discovered [22], [23].MiR-K12-11 was shown to mimic miR-155 function to induce a splenic B cell expansion in a NOD/SCID mouse model [24].

Investigating the combinatorial nature by which viral miRNAs expressed within a background of tissue-specific host miRNAs interact with their cognate transcriptomes requires genome-wide ribonomics-based approaches. Recently, high-throughput sequencing of RNA isolated by crosslinking immunoprecipitation (HITS-CLIP) and Photoactivatable-Ribonucleoside-Enhanced Crosslinking and Immunoprecipitation (PAR-CLIP)techniques have been developed that are based on the enrichment of Ago-miRNA-mRNA complexes from cells after UV cross-linking [25], [26]. While HITS-CLIP uses 254 nm UV light to cross-link RNA protein complexes, in PAR-CLIP cells are first treated with nucleoside analogs such as 4-thiouridine (4-SU) that are incorporated into nascent mRNAs, which are then cross-linked at 365 nm. After cross-linking, RNase treatment, and immunoprecipitation, small RNAs representing both miRNAs and their bound targets are extracted and converted into small RNA libraries that are analyzed by high-throughput sequencing. Very recently, Gottwein et al. reported a list of more than 2000 putative KSHV miRNA targets that were identified by PAR-CLIP in BC-1 and BC-3 cells [27]. Here we report on a detailed HITS-CLIP analysis of two commonly studied PEL cell lines, BCBL-1 and BC-3, which are both KSHV-positive but represent different B cell developmental stages [28], [29]. We identified 1170 and 950 genes, respectively, that were enriched for clusters of sequence tags containing KSHV miRNA seed sequence matches within 3′ UTRs and exons. Comparative analysis between both PEL cell lines revealed dramatic differences in Ago-associated miRNA repertoires and in the number and nature of miRNA targets, further supporting the idea that miRNA regulation can be highly cell type-and developmental stage-specific. In addition, comparison of our HITS-CLIP data with the PAR-CLIP data set reported by Gottwein et al. revealed about 42% overlap, which suggests that neither method enriches miRNA targets in a saturating manner. In summary, we have identified KSHV miRNA targets highly enriched for the gene ontology terms apoptosis, glycolysis, and lymphocyte activation, which will provide an important resource to further delineate the role of KSHV-encoded miRNAs for viral biology and pathogenesis.

## Results

### MiRNA and mRNA Ago HITS-CLIP library preparation from latently KSHV-infected PEL cells

**Figure 1:**
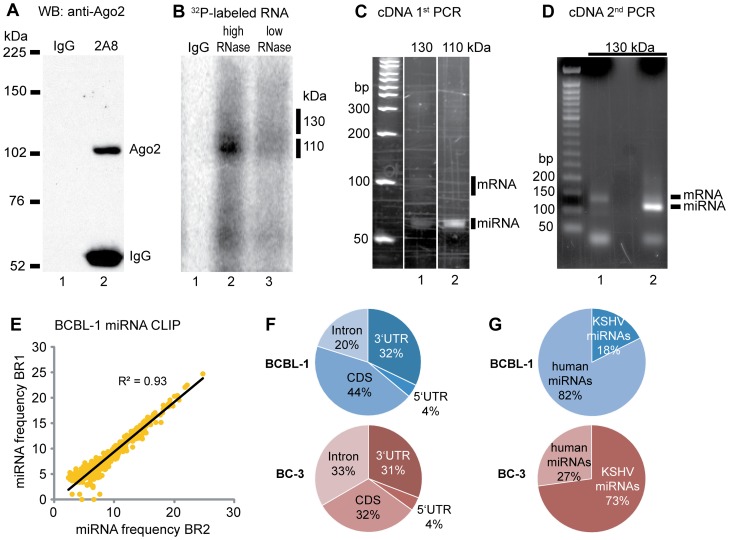
Ago HITS-CLIP in PEL cells. **A**: Western blot (WB) analysis of Argonaute immunoprecipitation with the anti-Ago antibody 2A8 [86] or an unspecific antibody (IgG) as control. The IP was blotted with the anti-Ago2 11A9 [88]. **B**: Autoradiogram of the radio-labeled RNA in cross-linked, immunoprecipitated Ago-miRNA-mRNA complexes after high or low RNAse treatment. Low RNase treatment yields a 110 and a 130 kDa complex. No complexes are visible with the control IgG. **C**: PCR products of the 1^st^ PCR amplification step separated by urea PAGE. Linker-ligated and reverse transcribed RNA extracted from the low RNAse treatment 110 kDa complex (B, lane 3) contained predominantly miRNAs (20–25 nt+∼40 nt linkers). The 130 kDa complex contained miRNAs and 50–70 nt mRNA tags (90–110 nt including linkers). MiRNA and mRNA PCR products of the 130 kDa complex shown in lane 1 were extracted for the 2^nd^ PCR amplification. **D**: products of the 2^nd^ PCR separated on an agarose gel. The longer primers used in this PCR amplification step yielded ∼115 bp products for the miRNAs and 140–160 bp products for the mRNAs. **E**: Scatter plot showing the correlation of the miRNA CLIP tags between biological replicates (BR) in BCBL-1 (shown as log_2_ of the miRNA frequency; for reproducibility in BC-3 see Figure S1B). **F**: distribution of mRNA-annotated clusters across transcripts. **G**: differential recovery of KSHV and human miRNAs associated with Ago in BCBL-1 and BC-3.

To identify genes that are targeted by KSHV and human miRNAs in latently KSHV-infected cells Ago HITS-CLIP was performed in the KSHV-positive and EBV-negative PEL cell lines BCBL-1 and BC-3.BCBL-1 cells are post germinal center B cells characterized by rearranged immunoglobulin loci [30]. BC-3 cells are pre-B cells, which have not undergone antigen-dependent B cell maturation [31]. As a result, both cell lines harbor significantly different transcriptomes [28], [29], [32]. HITS-CLIP was performed according to Chi et al. with minor changes of the immunoprecipitation (IP) and library construction protocols (for details see Materials and Methods and Text S1). IP of cross-linked and RNase-treated Ago-miRNA-mRNA-complexes from 1–2×10^8^ cells yielded two complexes migrating approximately at 110 kDa and 130 kDa (Figure 1A, B). While the smaller complex contained only short 20–25 nt long RNAs (presumably miRNAs), the 130 kDa complex contained two different RNA species: short RNAs (miRNAs) and 50–70 nt long RNAs (presumably target mRNAs) (Figure 1C, D). Both short and long RNA species derived from the 130 kDa complex were extracted and processed separately for library construction and deep sequencing (in the following referred to as miRNA libraries and mRNA libraries, respectively). To account for biological variance as observed in published HITS-CLIP data sets [25], [33]–[35] we performed three biological replicates for each cell line (BR1-3). As additional quality control one BCBL-1mRNA library(BR1) was sequenced in two technical replicates (TR1, 2). High throughput sequencing of six mRNA and five miRNA (2 from BCBL-1, 3 from BC-3) libraries was performed as 40 nts single strand runs and yielded more than 250 million sequence tags (16–31 million per run).To validate known and identify potential new host and viral miRNAs, sequence tags from miRNA libraries were aligned to miRBase(http:/mirbase.org/, release 17) using BLAST, and in addition analyzed using the miRDeep software package [36]. Nearly 90% of the miRNA library reads originated from human and KSHV miRNAs and comparison across BRs revealed a very high correlation of R^2^>0.92(Figure 1E and Figure S1A, and S1B). The comparison between BCBL-1 and BC-3 was lower(R^2^ = 0.65; Figure S1C), indicating significant differences in Ago-associated miRNA profiles betweenBCBL-1 and BC-3 as described in detail below.

Sequencing reads from all mRNA libraries were uploaded to the CLIPZ database, an open source software package specifically developed for the analysis of HITS-CLIP and PAR-CLIP data [37], and annotated to the human genome (hg19). The correlation for technical replicates was R^2^ = 0.88 (Figure S1D). Observed correlations across biological replicates (R^2^ = 0.53–0.72; Figure S1E, F) were comparable to previously reported HITS-CLIP data sets [25], [34].mRNA libraries were analyzed for clusters of overlapping reads using the CLIPZ sequence cluster tool. Of all the clusters aligning to mRNAs about two thirds were located in exons and one third in introns. Read distribution within exons largely reflected the current understanding of miRNA targeting, as the majority aligned to 3′UTRs and CDS (Figure 1F), and about 4% to 5′UTRs (7–8% after adjusting for possible UTR length bias; see Text S1). Read distribution within exons is also in agreement with recently published Ago HITS-CLIP and PAR-CLIP data sets [25]–[27], [34], [38], [39]. With respect to intron/exon distribution no significant differences were observed between mRNA libraries from BCBL-1 and BC-3 cells (Figure 1F and Figure S2).

### Profiles of Ago-associated miRNAs are markedly different in BCBL-1 and BC-3

**Figure 2:**
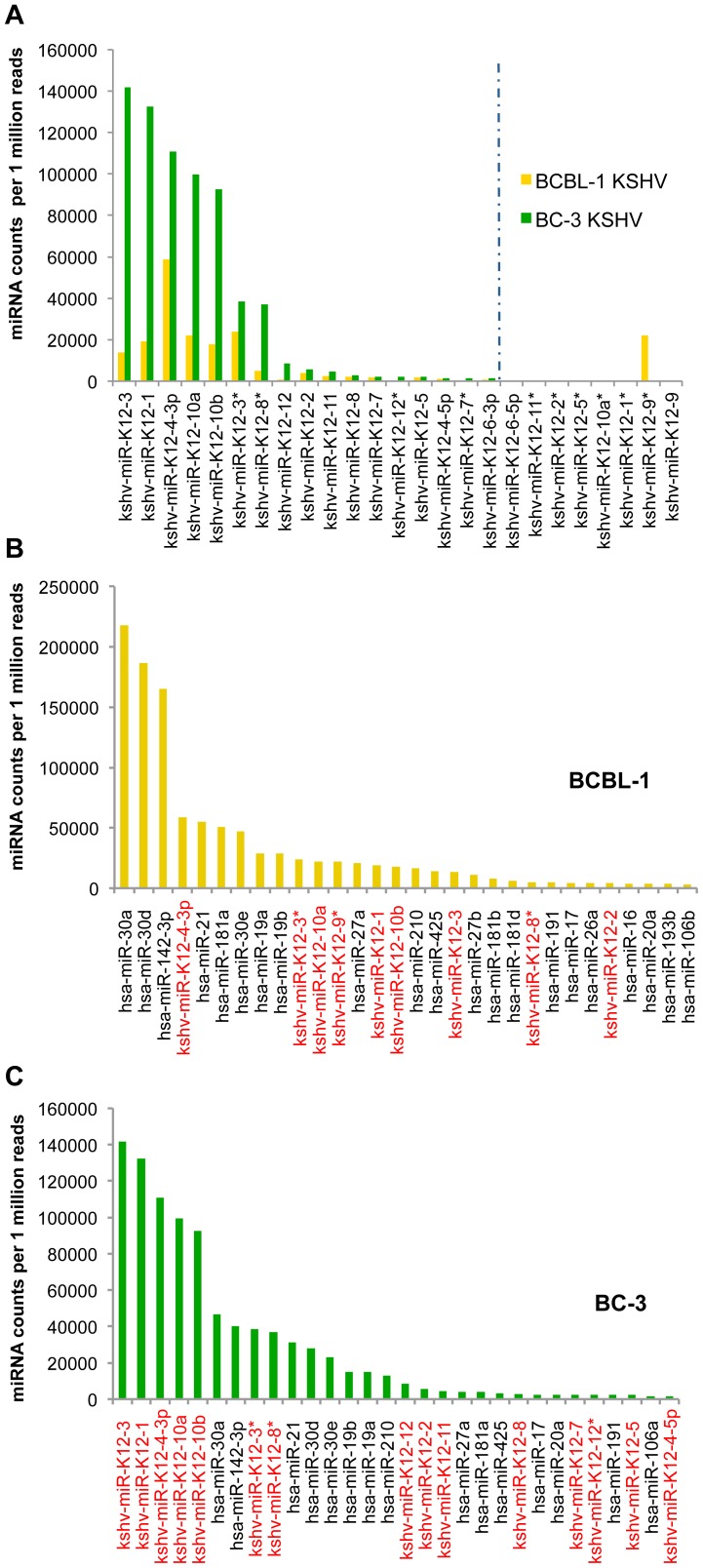
Ago-associated miRNA profiles in BCBL-1 and BC-3 cells. Shown are the normalized read counts for individual miRNAs recovered from 130 kDaAgo-miRNA-mRNA complexes. Counts were normalized to the total sequencing read numbers in the sample, rescaled to 1×10^6^ sequences (as a standard sample size) and then averaged over replicates. **A**: Comparison of KSHV miRNA reads in BCBL-1 and BC-3. The dashed line marks the cut-off set for the exclusion of miRNAs from targetome analysis (<200 read counts per 1×10^6^ sequencing reads). Note that miR-K12-9 and -9* counts were above the cut-off in BCBL-1 and therefore included in the targetome analysis. **B**: Top 30 miRNAs sequenced from the BCBL-1 miRNA libraries, **C**: Top 30 miRNAs sequenced from the BC-3 miRNA libraries.

A miRNA was counted as present if it was sequenced with at least one read in each BR and the average count over all BRs was at least 10. In BCBL-1,all 25 KSHV miRNAs were recovered, inBC-3 cells all except for miR-K12-9 and -9*,which are highly polymorphic and not expressed [12], [14]. However, we note that 7 KSHV miRNAs in both cell lines were detectable at very low read numbers (below 200 reads/million total reads; Figure 2A). We also detected 370 and 306 human miRNAs in BCBL-1 and BC-3, respectively. As observed previously by Chi et al. [25] the 30 most abundantly expressed miRNAs represent 94% of all miRNA reads (the top 20 contribute 90%), suggesting that only a small number of miRNAs act as major players in miRNA-mediated regulation of gene expression. A comparison of the miRNA libraries showed remarkable differences in the miRNA composition between the two PEL cell lines. While in BCBL-1 82% of the miRNA reads originate from human miRNAs, in BC-373% are KSHV-derived (Figure 1G). A more detailed analysis revealed that in BCBL-19 KSHV miRNAs rank within the top 30, with the most frequent one, miR-K12-4-3p, at position4. The three human lymphocyte-specific miRNAs hsa-miR 30a, 30d, and 142-3p occupy more than 50% of all RISCs in BCBL-1 cells(Figure 2B). In contrast, inBC-3 the top 5 miRNAs (miR-K12-3, -1, -4-3p, -10a, and 10b), as well as 15 of the top 30 miRNAs originate from KSHV (Figure 2C), contributing 74.5% of the top 30 and 71% of all miRNAs associated with Ago. At the same time, the read counts of miR-30a,-30d and miR-142-3p are dramatically decreased from 50% of all miRNA reads in BCBL-1 to 12% in BC-3. Also, individual viral miRNAs were associated with Ago at highly different frequencies in BCBL-1 and BC-3 cells. For example miR-K12-3, the most prevalent miRNA in BC-3, was 10-fold less abundant in BCBL-1 (Figure 2A).These results indicate that especially in BC-3 cells the KSHV miRNAs out-compete human miRNAs by displacing them from RISC complexes. In addition, we note that the pattern of human miRNA abundance differs between PEL cell lines, with some of the most abundant miRNAs in one cell line being found at much lower levels in the other. These abundantly expressed human miRNAs in BCBL-1 included miR-146, a major regulator of the inflammatory response [40], which was detected in BC-3 at almost 30-fold lower read numbers (data not shown). Vice versa, miR-155, whose aberrant expression is associated with multiple malignancies [41], [42] is not expressed in BCBL-1 but within the top 30 in BC-3. The differential Ago-association of KSHV and host miRNAs between these PEL cell lines suggests that marked differences may also exist in their respective miRNA targetomes.

### KSHV miRNA targetome analysis using the CLIPZ database

**Figure 3:**
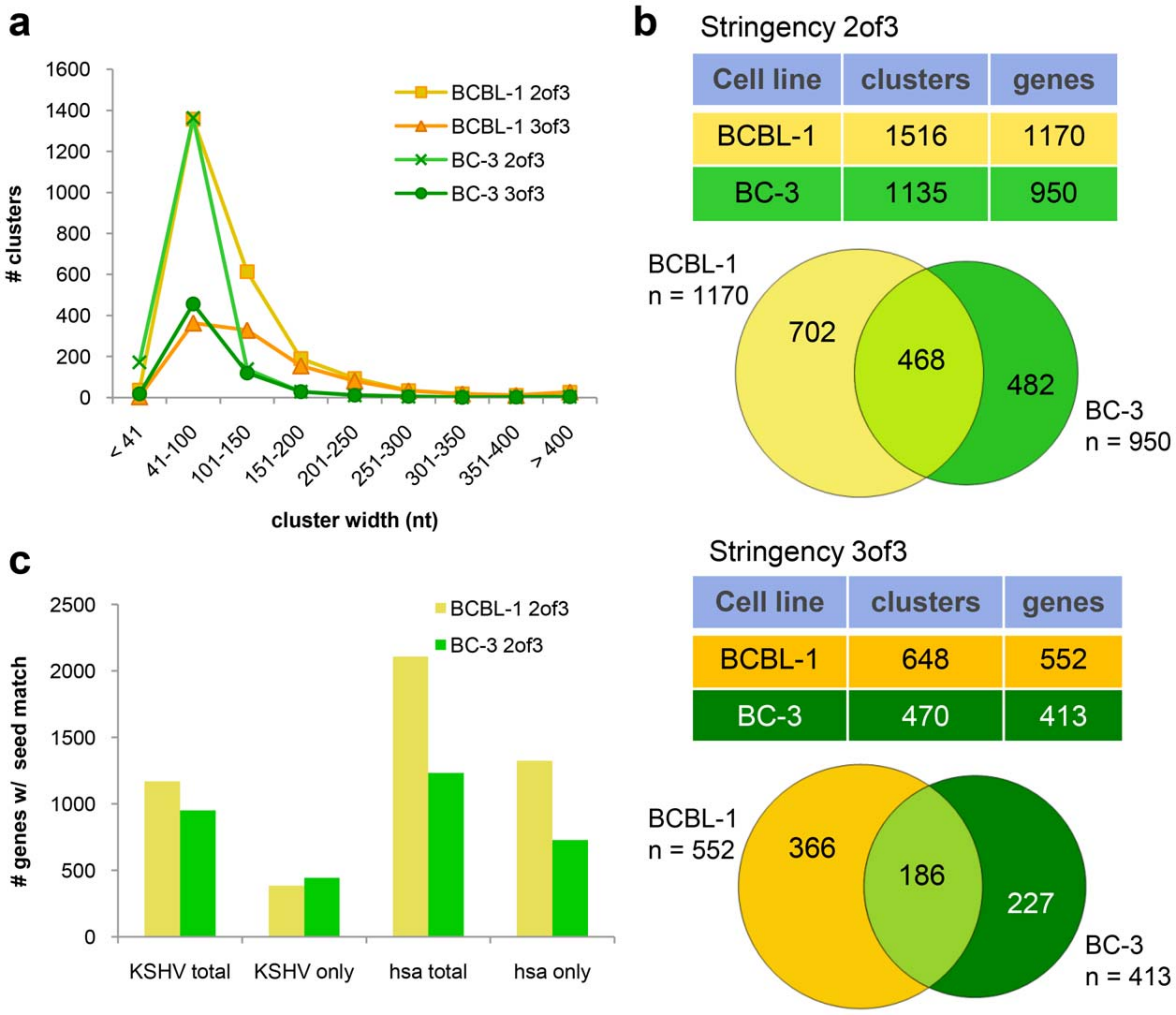
Analysis of sequencing read clusters obtained by Ago HITS-CLIP. mRNA libraries were analyzed for the presence of overlapping clusters of reads (super clusters) between BRs. Super cluster search was performed at two stringencies: i) super clusters present in 2 of 3 BRs (2of3), or ii) in all three BRs (3of3). The resulting super clusters were screened for miRNA seed match sites (nt 2–8) **A**: Cluster width distribution of super clusters containing any of the 25 (BCBL-1) or 23 (BC-3) KSHV miRNA seed matches; this analysis includes all super clusters aligning to annotated transcripts with a coverage of at least 2 copies/cluster per 10^6^ reads. Clusters listed multiple times due to different KSHV miRNA seed matches in the same cluster were counted only once. **B**: Number of potential KSHV miRNA targets identified by Ago HITS-CLIP for the top18 KSHV miRNAs in BCBL-1 and the top16 in BC-3. Counted are all clusters (and corresponding number of genes) in BCBL-1 and BC-3 that passed the filtering criteria in (A) and have a cluster width of 41–300 nt. Venn diagrams show the number of unique and overlapping genes between BCBL-1 and BC-3 that are targeted by KSHV miRNAs. **C**: Comparison of seed match occurrence for the top18 (BCBL-1)/top16 (BC-3) KSHV miRNAs and the top30 human miRNAs in BCBL-1 and BC-3. ‘KSHV total’: all genes targeted either exclusively by KSHV miRNAs, or contain additional human miRNA seed matches; ‘KSHV only’: genes targeted exclusively by KSHV miRNAs; ‘hsa total’ and ‘hsa only’ correspondingly. Note that the number of genes targeted by KSHV miRNAs differs only slightly between BCBL-1 and BC-3 despite the strong overrepresentation of KSHV miRNAs associated with Ago in BC-3 (see Figure 1G). The number of genes targeted by human miRNAs, however, is significantly higher in BCBL-1, reflecting the much higher number (and variety) of human miRNAs associated with Ago in BCBL-1.

For identification of putative miRNA targets, mRNA-derived clusters of overlapping reads were built on the human genome (hg19) within each BR followed by a search for overlapping clusters across BRs(superclusters). Super clusters were called at two different stringency criteria: clusters present in two of three BRs (stringency 2of3) or in all three BRs (3of3). Super clusters matching these criteria were then scanned for the presence of 7-mer seed matches (nt 2 to 8) of KSHV and the top 30 human miRNAs. Seed sequences for KSHV miRNAs that were recovered at very low frequencies (Figure 2A)were initially included in the search but not considered for the final target lists (for BCBL-1: miR-K12-6p, 11*, 2*, 5*, 10a*, and 1*; for BC-3 additionally miR-K12-9, 9*; for exclusion criteria see Text S1). Seed match-containing clusters were further filtered for alignment to annotated transcripts and sufficient coverage (for details see Text S1). Clusters that passed all filtering steps showed a tight width distribution of 41–150 nts (∼64% in BCBL-1 and 84% in BC-3), with more than 90% of all clusters being between 41 and 300 nts wide (Figure 3A and Table S1). The 41–300 nts wide, seed match-containing clusters, their associated genes, and targeting miRNAs identified at stringencies 2of3 and 3of3 were compiled into putative miRNA target lists for each cell line. We observed that clusters wider than 300 nts often consisted of overlapping peaks of different sizes, which didn't allow the identification of biologically meaningful seed pairing sites without individual visual inspection of each cluster. These clusters were therefore not included in the main target lists, but are listed as potential additional targets in separate tables. Prior to further analysis, we also asked whether target enrichment was correlated to transcript abundance, or biased by 3′UTR length and/or sequence composition. As expected, Ago HITS-CLIP recovered highly abundant transcripts with higher frequency(Table S2, Figure S3A and Text S1).With respect to 3′UTR length we found a weak bias towards longer 3′UTRs, however, short 3′UTRs (<300 bases) were enriched 5-fold higher than expected if enrichment would only be due to 3′UTR length instead of target specificity (Figure S3B).Finally, targets were enriched for genes with low GC content (Figure S3C), which may reflect less secondary structure and therefore better RISC accessibility. We note, however, that the overall variation in GC content across transcripts is moderate, with most transcripts being in the range of 35–55% GC.

BCBL-1 data (2of3) yielded 1516 clusters (41–300 nts wide) corresponding to 1170 transcripts, which contained one or more of the 18 included KSHV miRNA seed matches (Figure 3B). Using the highest stringency by calling clusters across all three BRs (3of3) yielded 648 clusters representing 552 transcripts. Stringency 2of3 inBC-3 yielded 1135 clusters (950 transcripts) targeted by 16 KSHV miRNAs, which was reduced to 470 clusters and 413 transcripts at the highest stringency (3of3). Comparing putative KSHV miRNA targets of both cell lines revealed that 50% or 468 of the transcripts targeted in BC-3 cells (2of3) were also targeted in BCBL-1 (Figure 3B). Complete target lists can be found in Tables S3 and S4.

Remarkably, despite the much larger number of KSHV miRNAs in BC-3 cells, the overall number of KSHV miRNA targets in the two cell lines is not very different and even lower in BC-3. Only the percentage of transcripts targeted exclusively by KSHV miRNAs is higher in BC-3 than in BCBL-1 (47 vs. 33% of all KSHV miRNA target transcripts, respectively; Figure 3C). Conversely, in congruence with the much higher levels of Ago-associated cellular miRNAs in BCBL-1, the overall number of transcripts containing human miRNA seed matches (with or without additional KSHV miRNA seed matches) as well as the number of transcripts exclusively targeted by host miRNAs was much higher in BCBL-1 than in BC-3(Figure 3C). In addition, the overall number of seed match-containing clusters and targets in BC-3 cells is smaller, which may be a result of the reduced miRNA complexity. These data show that the miRNA targetome in BC-3 cells is dominated by viral miRNAs.

**Figure 4:**
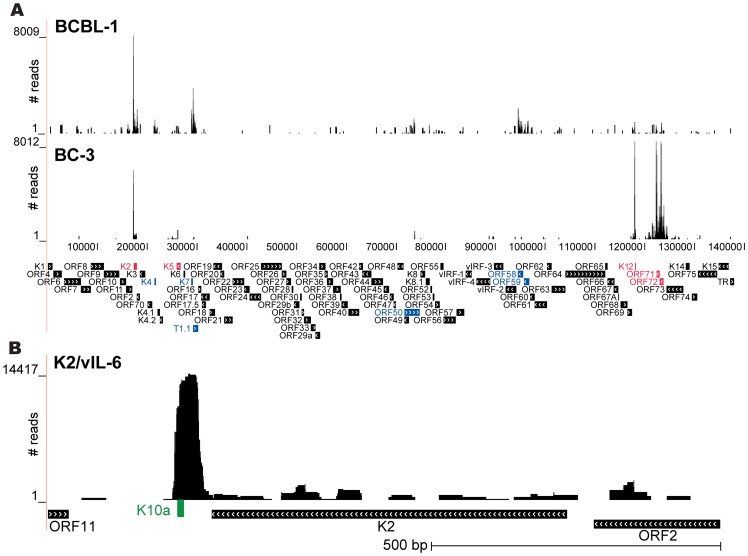
Ago HITS-CLIP clusters on the viral genome. **A:** Wiggle plots displaying read clusters across the KSHV genome in BCBL-1 and BC-3 cells. In contrast to BCBL-1, the KSHV genome is barely targeted in BC-3. Common targeted regions are the *K2/vIL-6* gene (predominantly its 3′UTR), *K5*,*K12/Kaposin*(3′UTR), the miRNA cluster region, *ORF71/vFLIP*, and *ORF72/vCyclin*. Reads were recovered at comparable numbers from *vIL-6* and *K5* in both cell lines, but with a much higher coverage from the other four common loci in BC-3. In BCBL-1 several additional potential target sites were detected (*K4*, *T1.1/PAN*, *ORF50/RTA*, *ORF58*, and *ORF59*). Major target sites in both cell lines are marked in red, sites targeted only in BCBL-1 are marked in blue; **B:** prominent read cluster in the 3′UTR of *K2/vIL-6* with a 6-mer miR-K12-10a target site.

A small proportion of the mRNA library reads, ranging from 0.15% to 1.05% (average 0.43%), originated from KSHV transcripts. Similar to miRNA expression levels and target numbers, these reads differed between both cell lines (Figure 4A). Overall, in BCBL-1more viral transcripts were enriched than in BC-3, which could be a result of the larger number of cellular miRNAs associated with Ago in BCBL-1, as described above. A prominent peak was present in both cell lines in the 3′UTR of *K2*, the viral *interleukin6* (*vIL-6*; Figure 4A, B), which is expressed in a subset of tumor cells at low levels during latency [43]. In addition, strongpeaks originated from the *K12/Kaposin* and *ORF71/vFLIP* 3′UTRs, and across the *vFLIP/vCyclin*(*ORF72*) transcripts (more prominent in BC-3 than in BCBL-1), as well as minor peaks at the miRNA cluster region and *K5*. Moreover, BCBL-1 showed additional peaks within *K4*, *T1.1/PAN*, *RTA/ORF50*, *ORF58*, and *ORF59* (Figure 4A).Within *ORF50*, some peaks were located within the open reading frame as well as downstream; we detected small clusters of reads over one of the potential miR-K12-5 binding sites [44] and over the miR-K12-9* target site [45] in the putative 3′UTR of *RTA*. Reads originating from the miRNA cluster likely represent the 1 to 2% miRNA reads recovered from the mRNA target libraries as well as pre-miRNA sequences [39]. We further validated the prominent peak within the 3′UTR of *vIL-6*, which contained a miR-K12-10a seed match by luciferase reporter assay as described below. While overall the enrichment of viral 7mer2-8 seed match-containing clusters was low across the viral genome, some ORFs and/or putative 3′UTRs contained clusters with host miRNA seed matches. Tracks showing enriched read clusters on the KSHV genome for all viral and the top 30 human miRNAs are provided in the supporting information (Dataset S1, S2, S3, S4).

### Ago HITS-CLIP in PEL cells identifies known and novel targets of KSHV miRNAs

**Figure 5:**
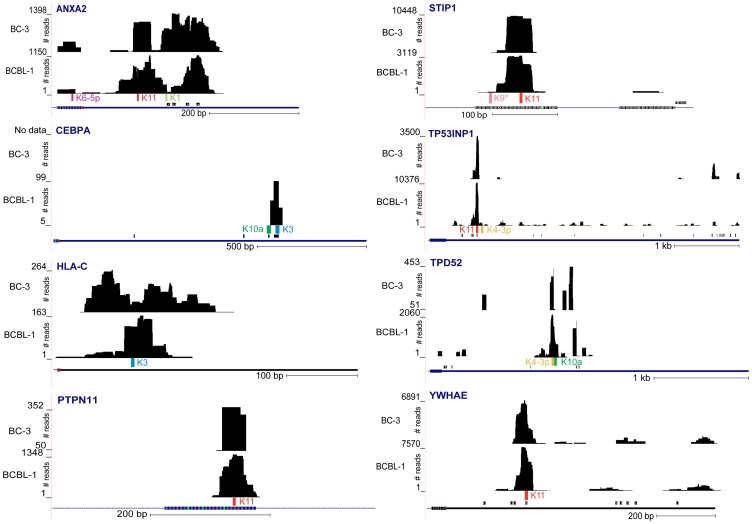
Ago-miRNA-mRNA clusters in new KSHV miRNA targets identified by Ago HITS-CLIP. mRNA-derived clusters of reads are visualized in UCSC genome browser as wiggle tracks. Shown are the positions of read clusters overlapping with miRNA seed match sites within 3′UTRs and exons of target transcripts in BCBL-1 and BC-3. KSHV miRNA seed match positions are indicated by colored bars; those of human miRNAs as predicted by TargetScan (for 3′UTRs only) are shown as black bars. Note that in BCBL-1 the major peak of TPD52 with the two KSHV miRNA seed match sites overlaps with several smaller peaks, resulting in a total cluster width of 340 nts. Therefore, for BCBL-1, TPD52 is listed in the ‘wide cluster’ list (Table S3C).

As a first validation of the target data set, we analyzed the read distribution over seed matches of31 experimentally confirmed KSHV miRNA targets reported by multiple groups [18], [19], [21]–[24], [44], [46]–[52]. From these, 16 were enriched by Ago HITS-CLIP and all but two showed enriched read clusters harboring the experimentally confirmed seed match (Table S5A).Some transcripts contained additional clusters with seed matches for other viral miRNAs. Figure S4 shows the read distribution of eight previously characterized targets visualized as wiggle plots in the UCSC genome browser. The target interactions of miR-K12-11, an ortholog of the oncomir miR-155 [22], [23], with *BTB and CNC homology 1, basic leucine zipper transcription factor 1* (*BACH1*), *Src-like-adaptor* (*SLA*), *FBJ murine osteosarcoma viral oncogene homolog* (*FOS*), and *CCAAT/enhancer binding protein beta* (*C/EBPβ*) have been confirmed by 3′UTR mutagenesis [22]–[24]. The 3′UTR of *BACH1* contains four, *FOS* and *SLA* each contain two, and *C/EBPβ* one seed match for miR-K12-11. Three of the *BACH1* sites previously demonstrated to be important for miR-K12-11 targeting were indeed enriched by HITS-CLIP; for the other three transcripts all miR-K12-11 seed match sites were occupied by clusters, although sometimes only in one biological replicate. Further comparison of recovered miR-K12-11 targets with a list of 151 putative miR-155 targets reported by multiple groups [22], [23], [53]–[61] revealed 30 commonly targeted transcripts (Table S5B). Dolken et al. reported on114putative KSHV miRNA targets that were enriched using immunoprecipitation in the absence of cross-linking (RIP-CHIP) [46]. Of these, 33 overlap with our data set including *NHP2 non-histone chromosome protein 2-like 1* (*NHP2L1*) and *leucine rich repeat containing 8 family, member D* (*LRRC8D*), which both recovered high frequency clusters for the validated miR-K12-3 target sites (Figure S4 and Tables S5A, S6A). Also, *Thrombospondin1*(*THBS1*) was previously shown to be targeted by multiple KSHV miRNAs [19]. Correspondingly, the HITS-CLIP data revealed seed match-containing clusters for miR-K12-1, -3, -3*, -6-3p, and -11. We note that all of the previously reported target sites for *THBS1* and *LRRC8D* consist of a 6-mer seed match and are therefore not included in the overall target lists(Table S3 and S4), but could be confirmed by manual investigation of the seed match sites(Figure S4). Figure 5 shows the read distribution for eight newly identified targets: *Annexin A2* (*ANXA2*), *CCAAT/enhancer binding protein alpha* (*C/EBPα*), *major histocompatibility complex, class I, C* (*HLA-C*), *protein tyrosine phosphatase, non-receptor type 11* (*PTPN11*), *stress-induced-phosphoprotein 1* (*STIP1*), *tumor protein p53 inducible nuclear protein 1* (*TP53INP1*), *tumor protein D52* (*TPD52*), and *tyrosine 3-monooxygenase/tryptophan 5-monooxygenase activation protein, epsilon polypeptide* (*YWHAE*), and their corresponding KSHV miRNAs. Both targeting and miRNA-specificity for these transcripts were further validated by 3′UTR luciferase assays (see below). This initial target validation demonstrates that our experimental HITS-CLIP conditions in combination with stringent filtering of clusters across biological replicates yielded a reliable working list of putative targets for KSHV miRNAs in BCBL-1 and BC-3 cells. Very recently, Gottwein et al. reported more than 2000 putative KSHV miRNA targets that were identified using PAR-CLIP in BC-1 and BC-3 cells [27]. We found that 830 or 42% of the putative targets identified by PAR-CLIP in BC-3 were also enriched by Ago HITS-CLIP in at least one biological replicate from BCBL-1 and/orBC-3 (Table S6B).

### Experimental target confirmation by luciferase reporter assays and Western blot

**Figure 6:**
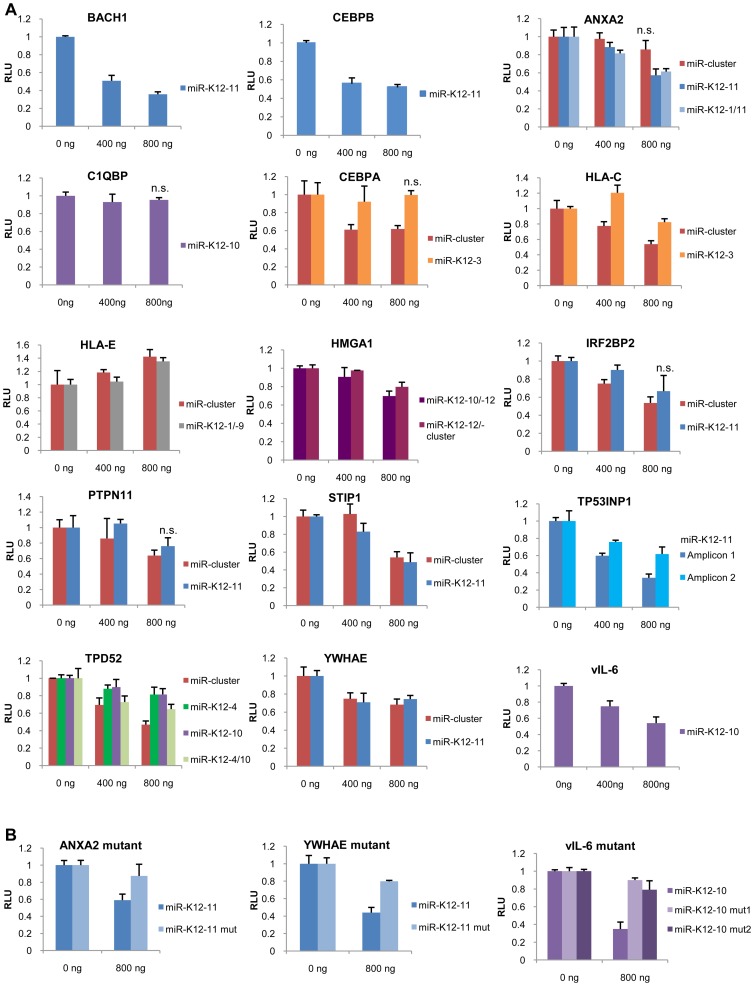
KSHV miRNA target validation by Luciferase Reporter Assay. **A**) 3′UTR or coding sequences of new KSHV miRNA target genes suggested by Ago HITS-CLIP were cloned into a Firefly Luciferase reporter vector and co-transfected into HEK293 cells together with different amounts of the corresponding KSHV miRNA/miRNA-cluster expression vector (or the empty vector control) and a transfection control vector. Firefly signal was assayed 72 hrs post transfection (Figure S6) and normalized to the signal of the transfection control vector. Transfections were performed in triplicates. Error bars represent standard deviation of triplicates. Shown is one representative of ≥3 independent experiments. Significance of reporter vector repression at the highest miRNA expression vector dose (800 ng) compared to the empty vector control (0 ng) was tested by two-tailed, unpaired t-test. p<0.05 unless indicated (n.s.). Significance was not tested if RLU at 800 ng was higher than or equal to RLU at 0 ng. Out of 13 tested new targets 11 showed a dose-dependent repression of the Luciferase expression in the presence of the miRNA(s) that was significantly (p<0.05) different from the empty vector control. Previously validated targets *BACH1* [22], [23] and *C/EBPβ* [24] served as positive controls. RLU = relative light units. **B**) For three targets, *ANXA2*, *YWHAE*, and *vIL-6*, the seed match site was mutated (see Figure S5 and Table S8), which, in an independent series of experiments, lead to a de-repression of the luciferase signal.

**Figure 7:**
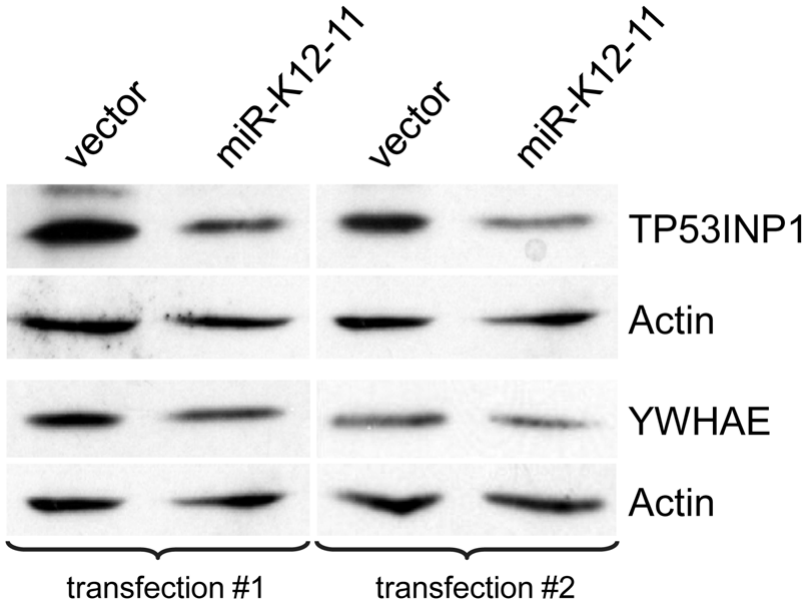
Western blot analysis of KSHV miRNA targets. Protein levels of two KSHV miRNA targets, TP53INP1 and YWHAE, were analyzed by Western blot in absence (0 ng) or presence (800 ng) of miR-K12-11 expression vector. Shown are two independent transfections of miR-K12-11. YWHAE showed moderate downregulation at the protein level, while TP53INP1 levels were reduced by more than 50% in the presence of miR-K12-11. Actin expression served as loading control.

Potential new KSHV miRNA targets were first aligned to their corresponding miRNA using RNA hybrid (Figure S5). We then cloned eight 3′UTRs and four enriched seed match-containing CDS downstream of a luciferase reporter cassette and performed miRNA sensor assays in HEK293 cells. For each transcript, we tested the predominantly identified miRNA or miRNA combinations and for some additionally the miRNA cluster, which contains 10 of the 12 miRNA genes as previously reported [19], [23]. As positive controls, we used the known miR-K12-11 targets *BACH1* and *C/EBPβ* [23], [24]. All eight 3′UTRs responded to miRNA expression with a dose-dependent decrease of luciferase expression by at least 20% (Figure 6A).These included *ANXA2*, *C/EBPα*, *HLA-C*, *high mobility group AT-hook 1* (*HMGA1*), *interferon regulatory factor 2 binding protein 2* (*IRF2BP2*), *TP53INP1*, *TPD52*, and *YWHAE*. We moreover introduced three point mutations in the miR-K12-11 seed match sites in the 3′UTRs of *ANXA2* and *YWHAE* (Figure S5). This resulted in a de-repression of both luciferase reporter constructs, thus further confirming the functionality of these target sites (Figure 6B). Finally, we showed by Western blot analysis a decrease of the TP53INP1 and YWHAE protein levels in the presence of miR-K12-11 (Figure 7).Of the four transcripts enriched for CDS seed matches, *PTPN11* and *STIP1* responded to miRNA expression while *HLA-E*, and *complement component 1, q subcomponent binding protein* (*C1QBP*) did not. This is in congruence with the literature reporting that miRNA target sites located within exons are less often functionally active [62]–[65]. Overall, 10 out of 12 putative targets were functionally confirmed. In addition, we tested the 3′UTR of *vIL-6*, which revealed a strong cluster that contained a 6-mer miR-K12-10a seed match. The *vIL-6* reporter was inhibited in a dose-dependent manner up to40% in the presence of miR-K12-10(Figure 6a). This effect was abolished by the introduction of two different miR-K12-10a seed match mutations (Figure 6B, S5). These data functionally confirm the first KSHV latency-associated gene to be modulated by a viral miRNA. Other putative viral targets including *RTA*, *vFLIP*, *vCyclin* and *Kaposin* are currently under investigation.

### Gene Ontology analysis of KSHV miRNA targets

All HITS-CLIP-derived KSHV miRNA targets found at analysis stringency 3of3 were subjected to Gene Ontology (GO) analysis using DAVID [66], [67]. GO analysis was performed against two different backgrounds: (i) the published BCBL-1 and BC-3 transcriptomes [32] and (ii) all human transcripts. Pathway enrichment was analyzed for five target subsets: all targets enriched in each cell line, cell line-specific targets, and overlapping targets between cell lines. A partial representation of enriched GO terms is shown in Table 1, the detailed GO analysis in Table S7. Genes involved in highly regulated processes often have long 3′UTRs and thus potentially contain more miRNA target sites. We therefore tested if GO terms were identified due to a bias for 3′UTR length rather than a functional enrichment. However, GO terms for three highly regulated processes (apoptosis, cell cycle, and glycolysis) showed only very moderate association with intermediate 3′UTR length and no association with long 3′UTRs (Figure S3D).

**Table 1:** Selected cellular pathways enriched in the KSHV miRNA targetome.

Commonly targeted pathways	# genes	KSHV miRNA targets identified by Ago HITS-CLIP
Regulation of apoptosis	42	YWHAZ, APH1A, EIF5A, PRDX2, HSPA1A, HSPA1B, ITM2B, CALR, FEM1B, PTEN, IL10, RPS3, SERINC3, SQSTM1, PPP2CA, PPP2CB, RAC1, TPT1, RHOA, RPL11, HSPA5, DYRK2, TOP2A, RPS27A, ARHGDIA, HSPA9, TXNIP, IL2RB, ARHGEF2, LGALS1, PIM2, RPS6, SOD1, YWHAE, NCSTN, SON, TNFRSF10B, EIF5AL1, CFL1, HSPB1, HSPD1, APBB1, UBA52, DNM2
Glycolysis	11	ALDOA, GPI, TPI1, LDHA, PKM2, PGAM1, HK2, PFKM, PGK1, GAPDH, MDH2
protein modification by small protein conjugation or removal	12	COPS5, TMEM189-UBE2V1, USP9X, UBE2V1, UBE3C, OS9, RBX1, UBE2D3, HUWE1, FBXO3, RPS27A, UBA52, FBXO11
protein modification by small protein conjugation	11	UBE2D3, TMEM189-UBE2V1, WWP2, SIAH1, UBE2V2, UBE3C, UBE2L3, RPS27A, RBX1, OS9, FBXO11
antigen processing and presentation	6	AP3D1, HLA-C, HLA-E, CALR, HLA-G

Pathways specific for BCBL-1	# genes	KSHV miRNA targets identified by Ago HITS-CLIP
lymphocyte activation	13	EGR1, BST2, MINK1, PRDX2, FKBP1A, MYH9, ITGB1, IL10, CD48, HSPD1, IRF4, DDOST, IMPDH1
regulation of I-kappaB kinase/NF-kappaB cascade	10	TNFRSF10B, TMEM189-UBE2V1, BST2, SLC20A1, SQSTM1, LGALS1, RHOA, UBE2V1, FKBP1A, PIM2, CANT1

Pathways specific for BC-3	# genes	KSHV miRNA targets identified by Ago HITS-CLIP
mitotic cell cycle	21	SEPT2, CKAP5, RAN, CAMK2G, TPX2, CCNG2, WEE1, NCAPD2, CDKN1A, RAD21, CDKN1B, CCND2, BCL2, FBXO5, PBRM1, KPNA2, CCNA2, STAG2, RPS27A, BUB3, STAG1
negative regulation of protein kinase activity	5	PPP2R1A, CDKN1A, CDKN1B, ZNF675, TRIB1
negative regulation of phosphate metabolic process	5	PPP2R1A, CDKN1A, CDKN1B, YWHAB, PEBP1

Enrichment was tested with all KSHV miRNA targets found at the stringency 3of3 using the Gene Ontology database DAVID [66], [67]. For complete results see Table S7.

### Putative KSHV miRNA targets are strongly enriched for pro-apoptotic factors and genes involved in glycolysis

In both cell lines Ago HITS-CLIP significantly enriched for KSHV miRNA targets involved in different pathways regulating apoptosis. Among the more than 40 genes were the *tumor necrosis factor receptor superfamily member 10b* (*TNFRSF10B*, miR-K12-1, -3) and the TP53 apoptosis effector *PERP* (miR-K12-3; p53 pathway), *FEM1B* (miR-K12-4-3p; Fas/TNFR1 signaling), and *Transforming Growth Factor beta Receptors* (*TGFBR*) *1* (miR-K12-2) and *3* (miR-K12-4-3p) (TGFBR pathway). The latter two proteins together with *growth factor receptor-bound protein 2* (*GRB2*, miR-K12-4-3p) also signal in the pro-apoptotic P70S6K pathway. Moreover, we identified the *tumor suppressor phosphatase and tensin homolog* (*PTEN*, miR-K12-4-3p, -7), a negative regulator of the anti-apoptotic Akt/PKB. Finally, we recovered several known apoptosis targets: *cyclin-dependent kinase inhibitor 1A* and *1B* (*CDKN1A* (miR-K12-11), *1B* (miR-K12-K12) [22], [27], which are also involved in cell cycle control, *Caspase 3* (*CASP3*), which was recently reported as miR-K12-1, -3, and -4-3p target [52], and *BCL2-associated transcription factor 1* (*BCLAF1*, miR-K12-2), which appears to have dual roles in PEL cells. While it was originally characterized as pro-apoptotic factor [68], [69], Ziegelbauer et al. found that in KSHV-infected cells BCLAF1 impairs apoptosis and also regulates lytic viral replication by sensitizing latent cells to reactivation stimuli [18].

The most enriched GO term in both cell lines was glycolysis (11 genes, p<4×10^6^). Recently, it was shown that KSHV infection of endothelial cells induces the Warburg effect during latency [70], which is observed in many human tumors and results in increased aerobic glycolysis and decreased oxidative phosphorylation [71]. Interestingly, initial experiments showed that latent KSHV infection of SLK cells leads to increased oxygen consumption (data not shown). However, testing 293 cells engineered to express the KSHV miRNA cluster containing 10 of the 12 miRNAs [19] failed to show a similar effect. Additional studies in SLK and primary endothelial cells are currently ongoing.

### Differential enrichment for genes involved in cell cycle control in BCBL-1 and BC-3 cells

In BCBL-1, Ago HITS-CLIP enriched for two inhibitors of the NFκB pathway, *lectingalactoside-binding soluble 1* (*LGALS1*, miR-K12-10b) [72] and *Interleukin 10* (*IL-10*, miR-K12-12*) [73]. This suggests that, in addition to positively regulating NFκB via the latency-associated vFLIP [74], KSHV further reinforces this crucial pathway for PEL cell survival by miRNA regulation. *IL-10*was also part of the GO term lymphocyte activation, which was highly enriched in BCBL-1-specific targets. We recovered 13 genes of this pathway including growth and/or differentiation factors such as *inosine 5′-monophosphate dehydrogenase 1*(*IMPDH1*, miR-K12-7), *early growth response 1*(*EGR1*, miR-K12-4-3p), *CD48* (miR-K12-7*), and *bone marrow stromal cell antigen 2* (*BST2*, miR-K12-8*).

In BC-3 cells, putative KSHV miRNA targets were enriched for factors that inhibit cell proliferation and G1 to S phase transition via multiple pathways. Among them were four inhibitors of Cyclin-dependent Kinase 2, including the previously characterized targets *CDKN1A* (p21; miR-K12-1) [22], *CDKN1B* (p27; miR-K12-11) and *Protein Phosphatase 2A* (*PP2A*; miR-K12-1) [27], as well as the *WEE1 homolog* (*WEE1*; miR-K12-1, -12). PP2A in addition causes G1 arrest via the BTG protein pathway. Moreover, p21 and p27 together with WEE1 block the progression of the cell cycle via E2F activation. We note that p21 and p27 expression is also regulated by vCyclin [75], [76]. The downregulation of these transcripts by KSHV miRNAs suggests a release of the cell cycle arrest and increased proliferation.

### Putative KSHV miRNA targets are involved in immune surveillance, ubiquitination, and negative regulation of kinase pathways

It was previously reported that KSHV miR-K12-7 targets *MHC class I polypeptide-related sequence B*(*MICB*) [47], which is also targeted by HCMV and EBV miRNAs [47], [77]. HITS-CLIP did not enrich for *MICB*, which might be expressed at levels too low to detect in PEL cells. However, HITS-CLIP enriched for KSHV miRNA targets involved in antigen presentation in the context of cellular immunity, i.e. the Major Histocompatibility (MHC) class I alpha-chain genes (*HLA-C*, *-E*, *-F*, and *–G*), and also genes involved in the process of loading and transporting MHC, i.e. *calreticulin* (*CALR*, miR-K12-4-3p, -10b) and *adaptor-related protein complex 3, delta 1* (*AP3D1*, miR-K12-3). We note that KSHV additionally encodes two E3 ubiquitin ligases, *ORF K3* (MIR1) and *OFR K5* (MIR2) that potently downregulate MHC I on the surface of infected cells [78], [79], suggesting another concerted action of KSHV miRNAs and proteins.

Several members of the ubiquitin conjugating (*TMEM189-UBE2V1*, miR-K12-1;*UBE2V2*, miR-K12-11; *UBE2L3*, miR-K12-1; and *UBE2D3*, miR-K12-2, -4-3p) and ubiquitin ligase (*UBE3C*, miR-K12-3) families were enriched in both cell lines, while negative regulators of kinases (e.g. *CDKN1A*, *CDKN1B*, and *PP2A*) were BC-3-specific (Table 1, Table S7). Potential signaling pathways modulated by these kinases and the question whether ubiquitin-dependent protein turnover is modulated by KSHV miRNAs needs to be experimentally addressed.

## Discussion

Performing Ago HITS-CLIP on BCBL-1 and BC-3 cells produced a catalogue of putative cellular and viral miRNA targets (Table S3, S4). We carefully modified the original Ago HITS-CLIP protocol [25] by adding more stringent wash steps(see Text S1) to the Ago immunoprecipitation. In addition, we constructed both the miRNA and mRNA libraries from the 130 kDa complex, while previous studies isolated miRNAs from the 110 kDa complex [25]. Three biological replicates and two technical replicates were sequenced to monitor biological variation and assay reproducibility. Finally, we excluded from the targetome analysis all KSHV miRNAs that were recovered at very low levels (Figure 2A). For the miRNA libraries, which are less complex, cross-validation was very high across biological replicates(R^2^>0.9; Figure S1A, S1B). For the mRNA libraries reproducibility was lower (R^2^≥0.53; Figure S1E, F) but well within the observed variation of previously published studies [25], [34]. Variability from the complex experimental procedure (see also below) and the small amount of Ago-associated RNA extracted and sequenced yields libraries that are not 100% representative; hence biological replicates add to both stringency and depths of the targetome analysis.

We retrieved 16 of 31 previously published KSHV miRNA targets (Table S5A) and recovered 33 of 114 genes identified in PEL cells by RIP-CHIP without prior UV cross-linking(Table S6A) [46]. Very recently, Gottwein et al. reported the identification of more than 2000 putative KSHV miRNA targets by PAR-CLIP [27]. While our study as well as the work from Gottwein et al. analyzed BC-3 cells, the remaining targets were defined in two very different cell lines, BCBL-1 (our study) and BC-1 (Gottwein et al.). BCBL-1 cells are infected only by KSHV, BC-1 cells also express a large number of EBV-encoded miRNAs. Moreover, Gottwein et al. allowed for 7mer1A seed matches, which were not included in our analysis. Comparison revealed42% overlapping targets between the PAR-CLIP BC-3 target list and our data set (BCBL-1 and BC-3; Table S6B). Moreover, enriched GO terms are similar between both studies. It is not well understood how both experimental platforms compare. Certainly, the cross-linking method (requirement for the presence of a uridine at the cross-linking position in PAR-CLIP, but not in HITS-CLIP), the nucleotide-specificity of the RNase used for clipping (RNase T1 vs. RNase A) and the extent of RNAse digest [34], as well as the choice of linkers (ligation bias; [80]), and finally the number of PCR cycles all contribute to differences in the composition of HITS-CLIP and PAR-CLIP libraries. Moreover, the experimental procedure contains two steps with a strong inherent variability: the excision of the Ago-miRNA-mRNA complexes, and the excision of the PCR products, which add substantial variation. On the bioinformatics side, the algorithms and parameters chosen for alignment to the reference genome, algorithms and cut-off criteria used for cluster calling, and definition of seed matches influence which targets will be present in the final target lists. To date, only one study directly compared HITS-CLIP and PAR-CLIP data sets and determined a cross-validation index of R^2^>0.4 to 0.65 between the two platforms [34], which is in good agreement with the overlap between our HITS-CLIP and the recent PAR-CLIP data [27].Notably, both data sets in the study by Kishore et al. [34] were analyzed by the same analysis pipeline. This suggests that PAR-CLIP and HITS-CLIP are both specific and that variations in the recovered miRNA targetome are mostly due to experimental rather than bioinformatics differences. To uncover the complete miRNA targetome may therefore require the combination of multiple approaches. Hence, the 1170 and 950 targets identified in 2 of 3 repetitions for BCBL-1 and BC-3, respectively, partially validate and moreover complement the PAR-CLIP data set, which lacked biological replicates [27].

### KSHV miRNAs may contribute to and reinforce the regulation of key pathways important for viral biology

The best characterized KSHV miRNA targets so far are mostly involved in regulating immune evasion (*MICB*), pro-apoptotic pathways (*BCLAF1*), and cell cycle control (*BACH1*, *FOS*, *THBS1*, *CDKN1A*, and *C/EBPβ*); for review see [7], [81]. The Ago HITS-CLIP-derived targetome shows strong enrichment for genes involved in these pathways, thus significantly expanding what to this point was solely based on single target gene studies. In addition, GO analysis suggests new host cell pathways to be targeted, such as glycolysis, lymphocyte activation and the ubiquitin/proteasome pathway, opening up additional interesting themes for functional studies. Finally, one clearly emerging concept from this HITS-CLIP data set is that multiple key pathways and processes such as the NFκB pathway, MHC class I-mediated immune surveillance, and cell cycle control can be co-regulated by both virally encoded proteins and miRNAs.

### BCBL-1 and BC-3 cells differ with respect to miRNA expression and targeting

MiRNA library analysis revealed strong differences in Ago-associated miRNAs in BCBL-1 and BC-3 cells, with KSHV miRNAs comprising 18% of all miRNA reads in BCBL-1, and an astonishing 73% in BC-3, and numbers of single KSHV miRNAs being up to 10-fold higher in BC-3. Similar results for the overall KSHV versus human miRNA count in both cell lines were obtained by the recent PAR-CLIP study [27]). Interestingly, several studies have analyzed KSHV miRNA expression in additional PEL cell lines and found differences not only with respect to overall expression levels but moreover also differences in the relative abundance of specific viral miRNAs [15], [82]. The fact that such expression differences likely affect targeting further supports the notion that miRNA targetomes are strictly context dependent.

Surprisingly, despite the much higher levels of KSHV miRNAs in BC-3 cells compared to BCBL-1, we identified similar KSHV miRNA target numbers in both cell lines, which were even 15–20% lower in BC-3. Only the number of transcripts exclusively targeted by KSHV miRNAs was slightly higher in BC-3 (Figure 3C). In contrast, we found that the number of genes targeted by human miRNAs (either exclusively or with additional KSHV sites), was almost 2-foldhigher in BCBL-1 than in BC-3. Thus, while the presence of more human miRNAs is correlated with more putative targets, the same appears to not be true for KSHV miRNAs. In this context it is interesting to note that we observed some differences between reported relative miRNA frequency observed by small RNA cloning [12], [27] and the relative frequency by which they were associated with Ago in BCBL-1 and BC-3 cells. Specifically, KSHV passenger strand miRNAs (miR-K12-3*, -5*, -8*, as well as -9* in BCBL-1), but also guide strand miRNAs (miR-K12-3, -10a, and 10b)are very modestly expressed, but have a relatively higher Ago-association rate (Figure S7). Moreover, for two of the three miRNAs with the highest incorporation-to-expression ratio and also an overall high incorporation level, miR-K12-3* and -8*, we identified only few targets. This raises the possibility of an additional function of some viral miRNAs besides seed sequence-specific target silencing: by being present in very high numbers in KSHV-infected PEL cells (especially in BC-3, but to a lesser extent also in BCBL-1as well as in BC-1 [27]), they might prevent human miRNAs from accessing RISCs, which would lead to a global de-repression of host genes. Indeed, we observed a strong impact on the target numbers of human miRNAs. Read counts of miR-142-3p and the miR-30 family, which are the most frequent Ago-associated miRNAs in BCBL-1, were reduced 4–5-fold in BC-3(Figure 2B, C). Accordingly, we also identified about 4-fold less targets in BC-3. Gene Ontology analysis showed that a significant fraction of the BCBL-1-specific miR-142-3p and miR-30 targets(many of them targeted by both miRNAs/families) are involved in protein transport and localization, chromatin organization, macromolecule catabolic processes, and protein degradation. Hence, these processes might be de-repressed in BC-3.Recent very elegant studies interrogating the quantitative aspects of miRNA targeting documented how shifting the ratio between miRNA and target mRNA copy numbers profoundly affects silencing efficiency [83], [84]. Hence, flooding host cells with viral miRNAs, a phenomenon first described by Dolken et al. in the context of *denovo* HCMV infection [85], maybe an additional mechanism by which herpesviruses induce cells into an activated state. Together with the fact that miRNAs from different viruses have evolved to target common pathways (i.e. apoptosis and cell cycle control) by direct silencing, this suggests that specific gene targeting and global inhibition of host miRNA function both contribute to gene expression differences in KSHV-infected cells.

In summary, our stringent and well-controlled approach provides a working list for functional follow-up studies to decipher viral (and host) miRNA function in KSHV-infected cells. In addition, the data strongly demonstrate that the KSHV miRNA targetome can significantly vary based on the miRNAs' overall abundance and RISC-incorporation, and by transcriptome differences between different PEL cell lines. As a consequence the putative PEL miRNA target catalogues presented by our HITS-CLIP data and the recently reported PAR-CLIP data [27] represent an important starting point for many mechanistic studies. However, a full understanding of the role that KSHV miRNAs play in viral biology will require the combination of viral genetics with ribonomics approaches performed in all cell types associated with KSHV pathogenesis as well as in primary tumor biopsies.

## Materials and Methods

### Ago HITS-CLIP

Ago HITS-CLIP procedure was performed in three biological replicates as described in Chi et al [25] with some minor modifications (for details see Text S1). Briefly, cells were harvested at a density of <0.8×10^6^ cells/ml and cross-linked at 254 nm prior to cell lysis. Ago-miRNA-mRNA complexes were immunoprecipitated from RNase-treated cross-linked lysates using the anti-Ago 2A8 antibody [86]. Immunoprecipitated RISC complexes were washed twice with cold high stringency buffer (15 mMTris-HCl, pH 7.5, 5 mM EDTA, 2.5 mM EGTA, 1% TX-100, 1% Na-deoxycholate, 0.1% SDS, 120 mMNaCI, 25 mM KCI), twice with high salt buffer (15 mMTris-HCl, pH 7.5, 5 mM EDTA, 2.5 mM EGTA, 1% TX-100, 1% Na-deoxycholate, 0.1% SDS, 1 M NaCI) [87], and then as described in Chi et al [25]. 130 kDaAgo-miRNA-mRNA complexes were separated by SDS-PAGE and RNA extracted from these complexes, yielding two different RNA species: short, 20–25 nt RNAs and longer, 50–70 nt RNAs. Both RNA species were treated as separate miRNA and mRNA libraries, respectively. RNA was reverse transcribed and PCR amplified for deep sequencing. Libraries were sequenced in 40 bp runs on an IlluminaGAIIx sequencer. miRNA libraries were analyzed using an in-house algorithm (see Text S1) and the software package miRDeep [36]. mRNA libraries were analyzed using the CLIPZ database [34]. Briefly, libraries were analyzed for overlapping reads (clusters), and then for overlapping clusters between biological replicates. Only clusters that overlapped at least between two biological replicates were considered for miRNA target search. These clusters were then analyzed for the presence of KSHV and human miRNA seed matches (nt 2–8).

### Gene Ontology analysis

Gene Ontology (GO) analysis was performed using the web-accessible database DAVID (http://david.abcc.ncifcrf.gov; [66], [67]) on KSHV miRNA targets found in all three biological replicates.

### Plasmids and Luciferase reporter assays

miRNA expression plasmids either contain a region of approximately 200 bp encompassing the pre-miRNA stem loop or the complete intronic miRNA cluster inserted into pcDNA3.1/V5/HisA [19]. Firefly luciferase reporter plasmids were created using the pGL3 promoter vector (Promega). Sequences of 3′UTRs or CDS were obtained from RefSeq. 3′UTRs were PCR amplified from BCBL-1 genomic DNA, CDS from BCBL-1cDNA, and cloned into the pGL3 promoter vector by GeneArt Seamless Cloning (Invitrogen) downstream of the Luciferase gene between the XbaI and the FseIsites.HEK293 cells were transfected with 2 ng of pCMV-Renilla control vector (Promega), 20 ng of the Firefly pGL3 reporter construct and 0, 400 or 800 ng of the pcDNA3.1 miRNA expression vector. The different concentrations of pcDNA3.1 miRNA expression vector were complemented with empty pcDNA3.1 vector to reach a total of 800 ng in each transfection. Cells were harvested 72 hrs post transfection and luciferase activity was quantified using the Promega Dual Luciferase Reporter kit according to the manufacturer's protocol.

### Western blotting and antibodies

Immunoblotting was carried out to detect down-regulation of miRNA targets at the protein level. Cell lysates were the same as used for luciferase reporter assays. 10–12 ug of total protein per lane were separated on 10% or 12% SDS gels and transferred to PVDF membranes using standard procedures. Membranes were probed with the following antibodies: rabbit anti-TP53INP1 (eBioscience, 14-6049), rabbit anti-YWHAE (Thermo Scientific, PA5-17104)), goat anti-actin-HRP (Santa Cruz, sc-1616), and goat anti-rabbit-HRP (Jackson Immunoresearch, 111-036-047)

Raw data have been uploaded to the CLIPZ database (www.clipz.unibas.ch) under the group name ‘Renne CLIP’ and are freely available for analysis and comparison with other CLIP data sets in the CLIPZ database and for download.

## Supporting Information

Dataset S1 **Wiggle track of all sequencing reads/clusters aligning to the KSHV genome in BCBL-1 cells.** For upload to UCSC Genome Browser. Sequencing reads were aligned to the KSHV RefSeq genome nc_009333.1. Aligned sequence files were converted to pileup and then to wiggle files. This track is the sum over the reads from all BCBL-1 BRs. Note that the track pretends alignment to position 1–137,950 of human chromosome 1 because UCSC Genome Browser does not provide the KSHV genome sequence. Therefore, the sequence displayed after upload to UCSC Genome Browser is not the sequence of the KSHV genome. The indicated position of the reads/clusters, however, reflects their true position on the KSHV genome. Note that if the user experiences problems with the upload to UCSC Genome Browser we recommend changing the file extension from ‘.txt’ to ‘.wig’.
(TXT)

Dataset S2 **Wiggle track of all sequencing reads/clusters aligning to the KSHV genome in BC-3 cells.** For upload to UCSC Genome Browser. Sequencing reads were aligned to the KSHV RefSeq genome nc_009333.1. Aligned sequence files were converted to pileup and then to wiggle files. This track is the sum over the reads from all BC-3 BRs. Note that the track pretends alignment to position 1–137,950 of human chromosome 1 because UCSC Genome Browser does not provide the KSHV genome sequence. Therefore, the sequence displayed after upload to UCSC Genome Browser is not the sequence of the KSHV genome. The indicated position of the reads/clusters, however, reflects their true position on the KSHV genome. Note that if the user experiences problems with the upload to UCSC Genome Browser we recommend changing the file extension from ‘.txt’ to ‘.wig’.
(TXT)

Dataset S3 **Bed file with the locations of the KSHV and top30 human miRNA 7mer2-8 seed matches on both strands of the viral genome.** For upload to UCSC Genome browser together with DatasetS1 and S2. Note that the track pretends alignment to position 1–137,950 of human chromosome 1 because UCSC Genome Browser does not provide the KSHV genome sequence. Therefore, the sequence displayed after upload to UCSC Genome Browser is not the sequence of the KSHV genome. The indicated position of the miRNA seed matches, however, reflects their true position on the KSHV genome. Note that if the user experiences problems with the upload to UCSC Genome Browser we recommend changing the file extension from ‘.txt’ to ‘.wig’.
(TXT)

Dataset S4 **Bed file with the locations of the KSHV ORFs and miRNA genes in the viral genome.** For upload to UCSC Genome browser together with DatasetS1 and S2. Coordinates of KSHV ORFs were extracted from the annotation provided by nc_009333.1.Note that if the user experiences problems with the upload to UCSC Genome Browser we recommend changing the file extension from ‘.txt’ to ‘.wig’.
(TXT)

Figure S1 **Reproducibility of the BCBL-1 and BC-3 miRNA and mRNA CLIP.** **A**)–**C**) miRNA libraries: miRNA read counts were normalized to the total sequencing read numbers in the sample and rescaled to 1×10^6^ sequences, which was chosen as standard sample size. The correlation between biological replicates (BR) was plotted as log_2_ of the miRNA frequency. **A**: BCBL-1, only two miRNA libraries were sequenced. **B**: BC-3, all three BRs were sequenced; **C**: correlation of miRNA frequencies between BCBL-1 and BC-3 (average over all BRs). **D**)–**F**) mRNA libraries: the agreement between the two technical replicates of BCBL-1 BR1 (**D**) and between biological replicates (**E, F**) of the mRNA libraries is shown as difference plots (Bland-Altman plot), which are a good method to examine the consistency among samples [89]–[92]. For each TR or BR, the coverage of reads in the super cluster regions (stringency 2of3 for BRs, and 2of2 for the two TRs) was quantified in reads per kilobase of exon model per million mapped reads (RPKM [93]). The RPKM values were calculated using an in-house Perl script. Plots were made in R. The scripts are available upon request. The absolute differences in RPKM values between two replicates (y axis; e.g. [BR2-BR1]) are plotted against the mean of the replicates (x axis; e.g. [BR1+BR2]/2). The red line indicates the mean difference, the green lines the mean difference plus and minus the standard deviation of the differences.
(TIF)

Figure S2 **Distribution of mRNA-annotated reads across transcripts.** Comparison of the percentage of mRNA-annotated reads aligning to 3′UTR, 5′UTR, CDS and intron, shown for the average over all replicates (top) and for individual replicates in BCBL-1 (left) and BC-3 (right).
(TIF)

Figure S3 **Ago HITS-CLIP targets are enriched for higher transcript frequency and lower GC content.** Human transcripts were sorted into 5 bins with equal number of genes according to their transcript frequency, GC content or 3′UTR length. Ago HITS-CLIP-identified targets of KSHV and human miRNAs were then separately associated with the bins and counted. We also calculated the expected relative target numbers in each bin if there was no association between the probability to identify a target and the target properties (frequency, GC content, 3′UTR length), shown as red bars, and the expected numbers in case of linear association (orange bars). **A**) Test for enrichment due to transcript frequency. **B**). Test for enrichment due to 3′UTR length. The x axis shows the average transcript length (nt) over all transcripts in each bin. For the range of transcript lengths in each bin see Table S2. **C**) Test for enrichment due to GC content. X axis shows the average GC content (%) in each bin. For the range of GC content in each bin see Table S2. **D**) Test for enrichment for three highly regulated GO terms, apoptosis, glycolysis and cell cycle, due to 3′UTR length. X axis shows the average transcript length (nt) over all transcripts in each bin.
(TIF)

Figure S4 **Ago-miRNA-mRNA clusters in known KSHV miRNA targets identified by Ago HITS-CLIP.** mRNA-derived clusters of reads are visualized in UCSC genome browser as wiggle tracks. Shown are the positions of read clusters overlapping with miRNA seed match sites within 3′UTRs and exons of target transcripts in BCBL-1 (*BACH1*, *THBS1*, *SLA*, *FOS*, *NHP2L1*, *LRRC8D*, and *CEBPB*) and BC-3 (*NFKBIA*). KSHV miRNA seed match positions are indicated by colored bars. Functionality of seed match sites was confirmed by Luciferase reporter assays and seed match mutations [19], [22]–[24], [46].
(TIF)

Figure S5 **RNAhybrid alignments between KSHV miRNAs and new targets.** RNAhybrid (http://bibiserv.techfak.uni-bielefeld.de/rnahybrid/) alignments were performed for all new targets confirmed by Luciferase reporter assays (see Figure 6). 7mer2-8 seed match sites are highlighted in yellow, mutated bases within the seed match are marked in red. RNAhybrid did not provide an alignment between miR-K12-3 and the 3′UTR of HLA-C, and between miR-K12-10a* and the 3′UTR of HMGA1.
(DOCX)

Figure S6 **Luciferase reporter assay time course.** To determine the optimal harvest time for monitoring miRNA-mediated reporter repression, a time course was performed with theBACH1 (miR-K12-11) and vIL-6 (miR-K12-10) luciferase reporter constructs. Transfections were performed as described and cells harvest at 24, 48, and 72 hrs post transfection and assayed for luciferase expression. The time course clearly shows the highest reporter repression for both targets at 72 hrs.
(TIF)

Figure S7 **MiRNA association with Ago proteins is not correlated with miRNA expression level.** MiRNA association with Ago as observed by Ago HITS-CLIP in BCBL-1 (**A**) and BC-3 cells (**B**) was plotted against miRNA expression levels as previously determined by small RNA cloning and deep sequencing [27]. All miRNA counts were normalized to the total miRNA sequencing reads obtained for each sample, rescaled to 1×10^6^ reads and plotted as log_2_ of the normalized read counts. KSHV miRNAs are shown as red dots, human miRNAs as blue dots. The centerline represents equal Ago-association and -expression ratio.
(TIF)

Table S1 **Cluster width distribution of KSHV miRNA seed match-containing clusters.**
(DOCX)

Table S2 **Target transcript abundance (A), target 3′UTR length distribution (B), and target 3′UTR GC content (C) in Ago HITS-CLIP identified miRNA targets.**
(DOCX)

Table S3 **Target genes of the top 18 KSHV miRNAs and the top 30 human miRNAs in BCBL-1.**
(XLSX)

Table S4 **Target genes of the top 16 KSHV miRNAs and the top 30 human miRNAs in BC-3.**
(XLSX)

Table S5 **Recovery of validated miRNA target genes by Ago HITS-CLIP.**
(XLSX)

Table S6 **Comparison of Ago HITS-CLIP data with published KSHV miRNA target lists.**
(XLSX)

Table S7 **Gene Ontology analysis.**
(XLSX)

Table S8 **Oligonucleotide sequences.**
(XLSX)

Text S1 **Supplementary methods and supplementary references.**
(DOCX)
