# Correction: Ago HITS-CLIP Expands Understanding of Kaposi's Sarcoma-associated Herpesvirus miRNA Function in Primary Effusion Lymphomas

**DOI:** 10.1371/annotation/bb6f7450-8506-41c3-8519-020df8b382b9

**Published:** 2013-03-28

**Authors:** Irina Haecker, Lauren A. Gay, Yajie Yang, Jianhong Hu, Alison M. Morse, Lauren M. McIntyre, Rolf Renne

The CLIPZ database will not allow downloading of the raw sequencing data for analysis and comparison. The entire data set has been uploaded to the NCBI Gene Expression Omnibus, where it is now listed under Accession number GSE41357. The data set can be found here: http://www.ncbi.nlm.nih.gov/geo/query/acc.cgi?acc=GSE41357

