# Supplementary figures and images for: Ago HITS-CLIP Expands Understanding of Kaposi's Sarcoma-associated Herpesvirus miRNA Function in Primary Effusion Lymphomas
Source: PLoS Pathog. 2012 Aug 23;8(8):e1002884. doi: 10.1371/journal.ppat.1002884 (PMC3426530; doi:10.1371/journal.ppat.1002884)

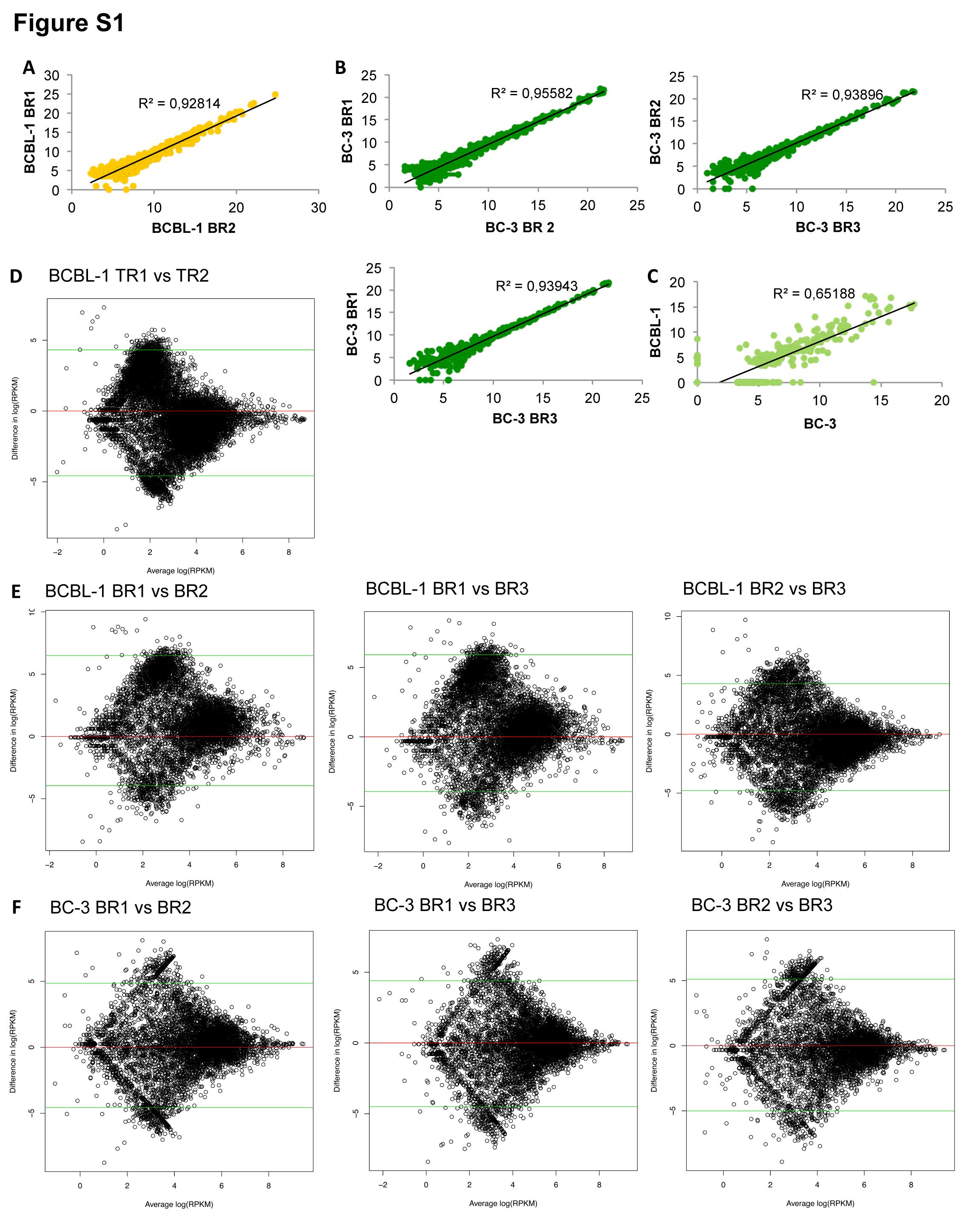

Supplement: Figure S1 — Reproducibility of the BCBL-1 and BC-3 miRNA and mRNA CLIP. A)–C) miRNA libraries: miRNA read counts were normalized to the total sequencing read numbers in the sample and rescaled to 1×10^6^ sequences, which was chosen as standard sample size. The correlation between biological replicates (BR) was plotted as log2 of the miRNA frequency. A: BCBL-1, only two miRNA libraries were sequenced. B: BC-3, all three BRs were sequenced; C: correlation of miRNA frequencies between BCBL-1 and BC-3 (average over all BRs). D)–F) mRNA libraries: the agreement between the two technical replicates of BCBL-1 BR1 (D) and between biological replicates (E, F) of the mRNA libraries is shown as difference plots (Bland-Altman plot), which are a good method to examine the consistency among samples [89]–[92]. For each TR or BR, the coverage of reads in the super cluster regions (stringency 2of3 for BRs, and 2of2 for the two TRs) was quantified in reads per kilobase of exon model per million mapped reads (RPKM [93]). The RPKM values were calculated using an in-house Perl script. Plots were made in R. The scripts are available upon request. The absolute differences in RPKM values between two replicates (y axis; e.g. [BR2-BR1]) are plotted against the mean of the replicates (x axis; e.g. [BR1+BR2]/2). The red line indicates the mean difference, the green lines the mean difference plus and minus the standard deviation of the differences. (TIF) [file ppat.1002884.s005.tif]

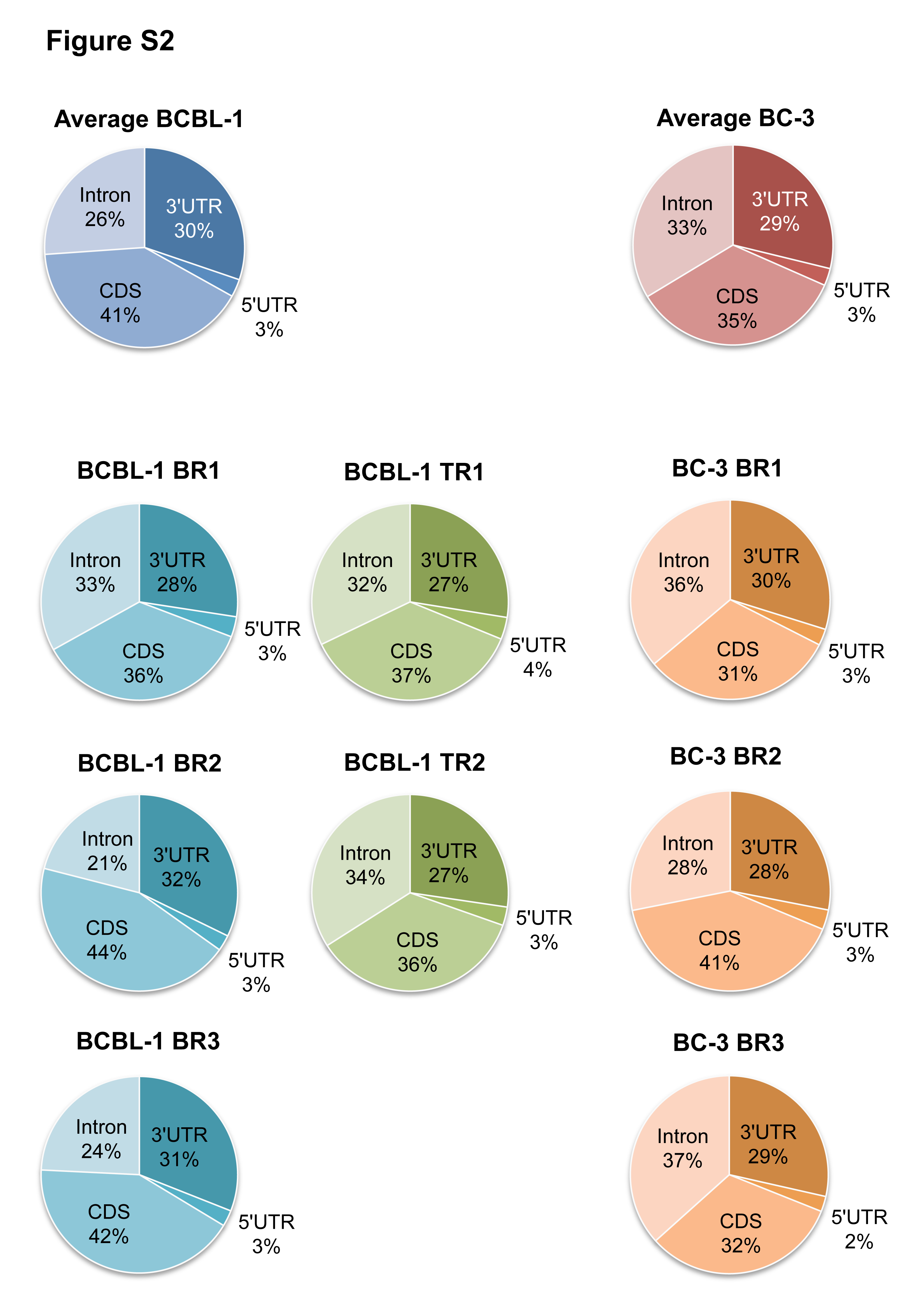

Supplement: Figure S2 — Distribution of mRNA-annotated reads across transcripts. Comparison of the percentage of mRNA-annotated reads aligning to 3′UTR, 5′UTR, CDS and intron, shown for the average over all replicates (top) and for individual replicates in BCBL-1 (left) and BC-3 (right). (TIF) [file ppat.1002884.s006.tif]

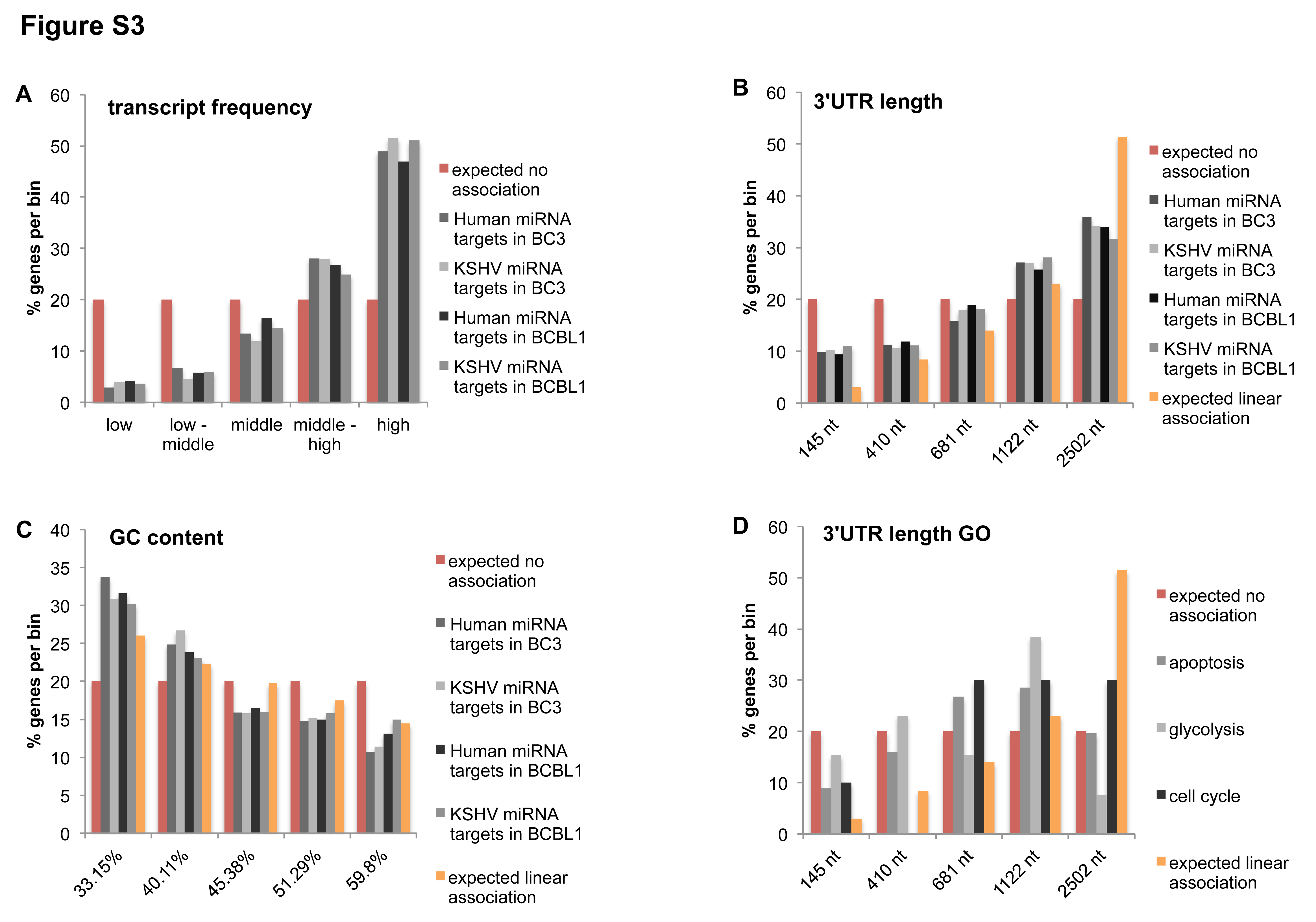

Supplement: Figure S3 — Ago HITS-CLIP targets are enriched for higher transcript frequency and lower GC content. Human transcripts were sorted into 5 bins with equal number of genes according to their transcript frequency, GC content or 3′UTR length. Ago HITS-CLIP-identified targets of KSHV and human miRNAs were then separately associated with the bins and counted. We also calculated the expected relative target numbers in each bin if there was no association between the probability to identify a target and the target properties (frequency, GC content, 3′UTR length), shown as red bars, and the expected numbers in case of linear association (orange bars). A) Test for enrichment due to transcript frequency. B). Test for enrichment due to 3′UTR length. The x axis shows the average transcript length (nt) over all transcripts in each bin. For the range of transcript lengths in each bin see Table S2. C) Test for enrichment due to GC content. X axis shows the average GC content (%) in each bin. For the range of GC content in each bin see Table S2. D) Test for enrichment for three highly regulated GO terms, apoptosis, glycolysis and cell cycle, due to 3′UTR length. X axis shows the average transcript length (nt) over all transcripts in each bin. (TIF) [file ppat.1002884.s007.tif]

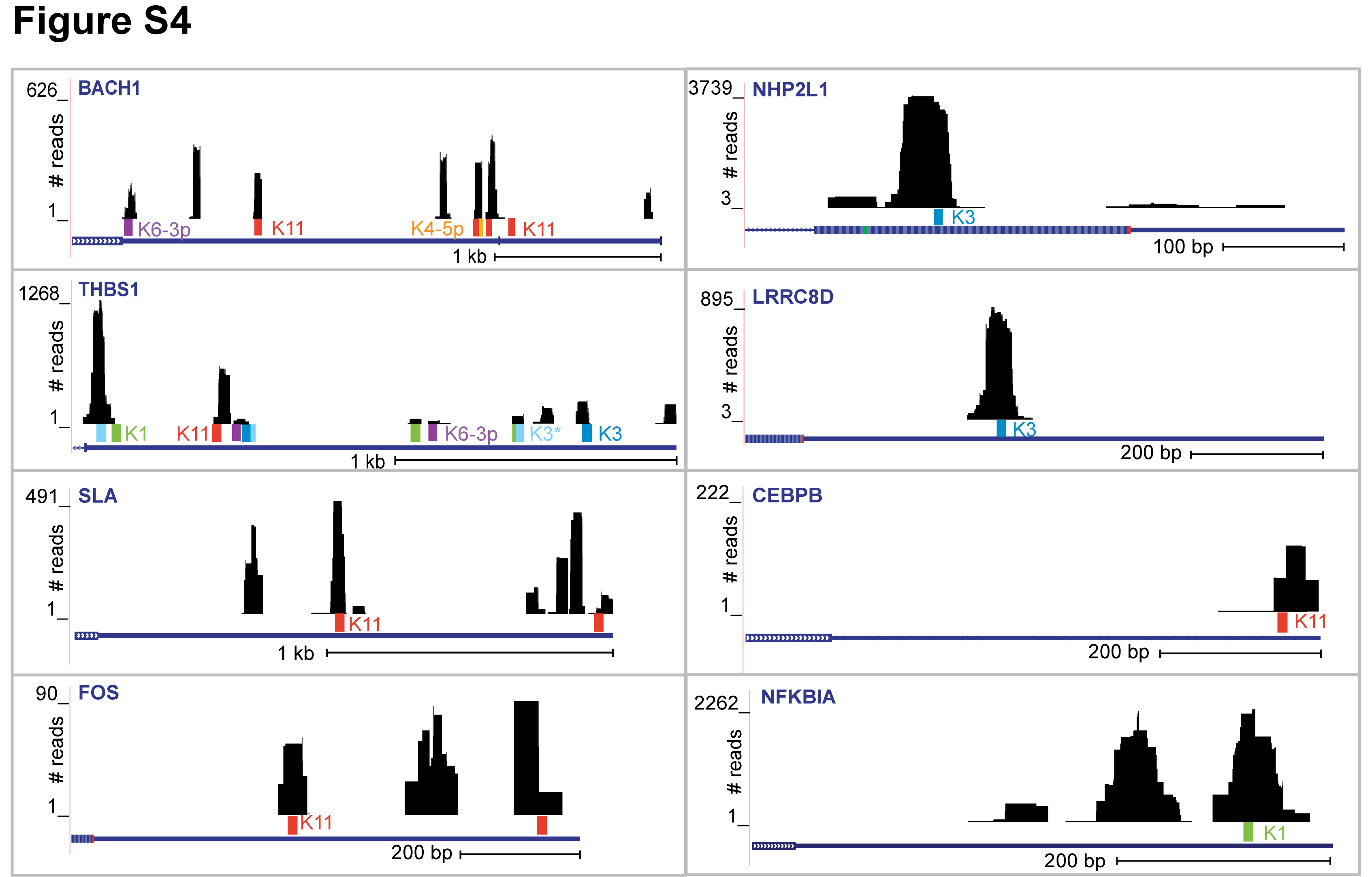

Supplement: Figure S4 — Ago-miRNA-mRNA clusters in known KSHV miRNA targets identified by Ago HITS-CLIP. mRNA-derived clusters of reads are visualized in UCSC genome browser as wiggle tracks. Shown are the positions of read clusters overlapping with miRNA seed match sites within 3′UTRs and exons of target transcripts in BCBL-1 (BACH1, THBS1, SLA, FOS, NHP2L1, LRRC8D, and CEBPB) and BC-3 (NFKBIA). KSHV miRNA seed match positions are indicated by colored bars. Functionality of seed match sites was confirmed by Luciferase reporter assays and seed match mutations [19], [22]–[24], [46]. (TIF) [file ppat.1002884.s008.tif]

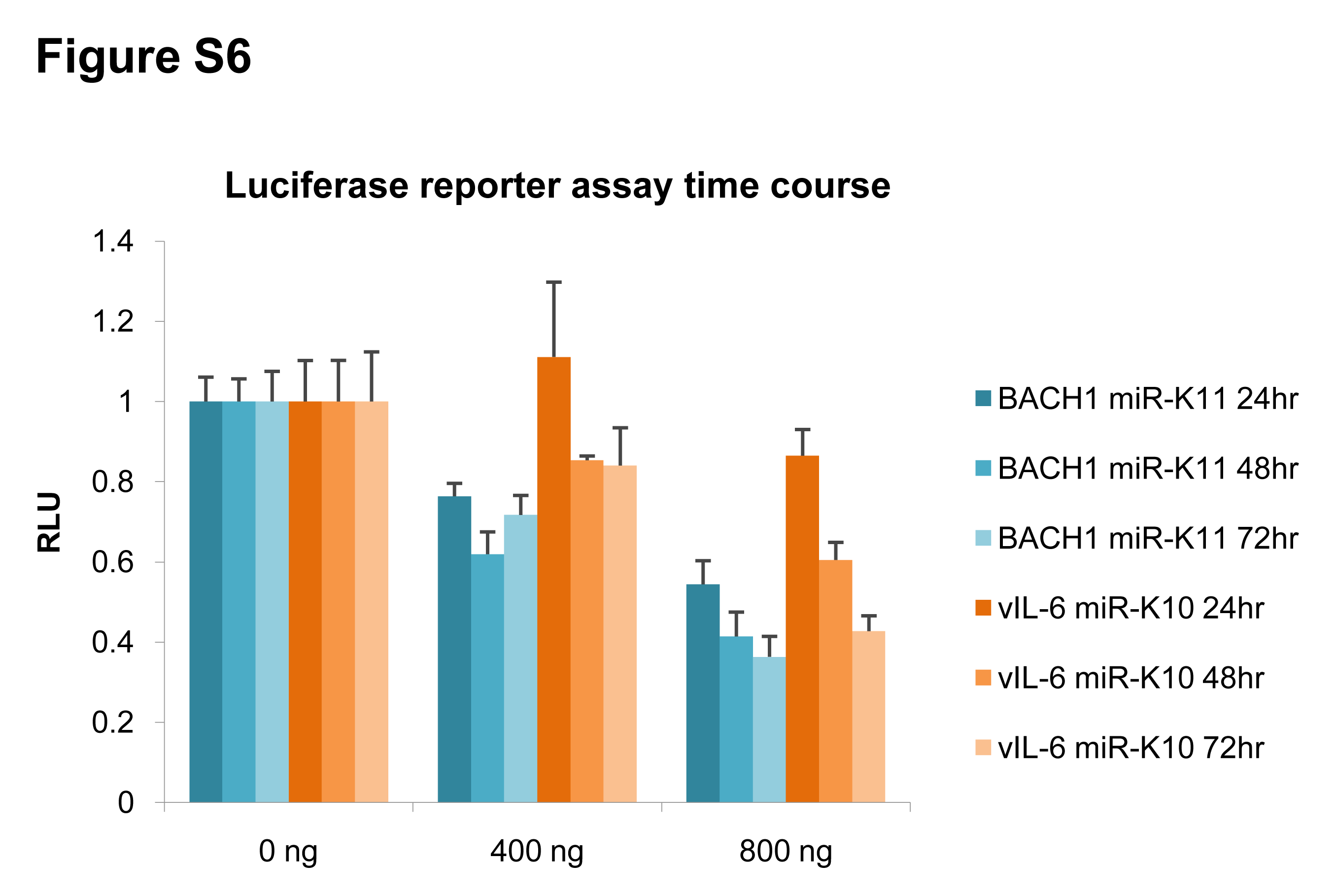

Supplement: Figure S6 — Luciferase reporter assay time course. To determine the optimal harvest time for monitoring miRNA-mediated reporter repression, a time course was performed with theBACH1 (miR-K12-11) and vIL-6 (miR-K12-10) luciferase reporter constructs. Transfections were performed as described and cells harvest at 24, 48, and 72 hrs post transfection and assayed for luciferase expression. The time course clearly shows the highest reporter repression for both targets at 72 hrs. (TIF) [file ppat.1002884.s010.tif]

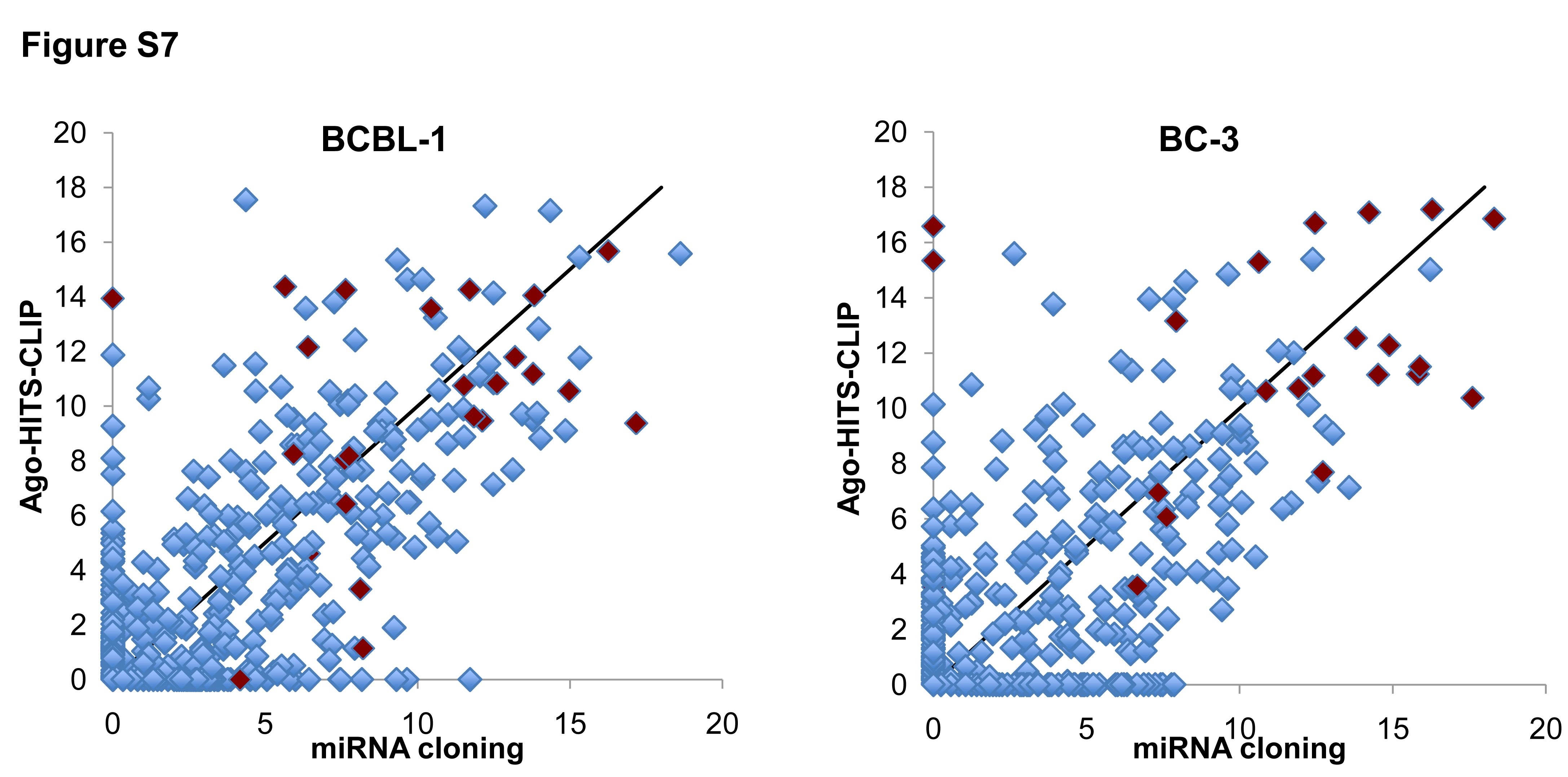

Supplement: Figure S7 — MiRNA association with Ago proteins is not correlated with miRNA expression level. MiRNA association with Ago as observed by Ago HITS-CLIP in BCBL-1 (A) and BC-3 cells (B) was plotted against miRNA expression levels as previously determined by small RNA cloning and deep sequencing [27]. All miRNA counts were normalized to the total miRNA sequencing reads obtained for each sample, rescaled to 1×10^6^ reads and plotted as log2 of the normalized read counts. KSHV miRNAs are shown as red dots, human miRNAs as blue dots. The centerline represents equal Ago-association and -expression ratio. (TIF) [file ppat.1002884.s011.tif]
