# Supplementary material for: Ago HITS-CLIP Expands Understanding of Kaposi's Sarcoma-associated Herpesvirus miRNA Function in Primary Effusion Lymphomas
Source: PLoS Pathog. 2012 Aug 23;8(8):e1002884. doi: 10.1371/journal.ppat.1002884 (PMC3426530; doi:10.1371/journal.ppat.1002884)
Supplement: Figure S5 — RNAhybrid alignments between KSHV miRNAs and new targets. RNAhybrid (http://bibiserv.techfak.uni-bielefeld.de/rnahybrid/) alignments were performed for all new targets confirmed by Luciferase reporter assays (see Figure 6). 7mer2-8 seed match sites are highlighted in yellow, mutated bases within the seed match are marked in red. RNAhybrid did not provide an alignment between miR-K12-3 and the 3′UTR of HLA-C, and between miR-K12-10a* and the 3′UTR of HMGA1. (DOCX) [file ppat.1002884.s009.docx]

**Figure S5**

**TARGET : ANXA2-3’UTR**

**MiRNA : K11**

target 5' G C CCCUGUGA GACGUU C 3'

UGG CAU GGGU AGCAUUA

GCC GUG UCCG UCGUAAU

miRNA 3' A U AU U 5'

**targetM** 5' G C CCCUGUGA GACGUU C 3'

UGG CAU GGGU AcgAUaA

GCC GUG UCCG UCGUAAU

miRNA 3' A U AU U 5'

**TARGET : ANXA2-3’UTR**

**MiRNA : K1**

target 5' A A CUG UCCUGUCU CUC G 3'

AGC UUGC GCCU AGU UCCUGUAA

UCG AAUG UGGG UCA AGGACAUU

miRNA 3' A A 5'

**TARGET : C1QBP-CDS**

**MiRNA : K10**

target 5' U ACCGA U U 3'

GCCG GGGG GGACAACACU

CGGU CCCC CCUGUUGUGA

miRNA 3' GAG U 5'

**TARGET : CEBPA-3’UTR**

**MiRNA : K3**

target 5' A AG UU G AA GU C 3'

GC UGCC GU C U GAAUGUG

CG ACGG CA G A CUUACAC

miRNA 3' CAG G GU U 5'

**TARGET : HLA-E-CDS**

**MiRNA : K9***

target 5' C AUGCAU GC G 3'

AGUGG G UGCG AGCUGGG

UCGCC C AUGC UCGACCC

miRNA 3' C AA G A 5'

**TARGET : HLA-E-CDS**

**MiRNA : K1***

target 5' A CC CCUG G 3'

UG AGG AC AGGUGCUG

AC UCC UG UCCACGAC

miRNA 3' CA G UU G 5'

**TARGET : HMGA1-3’UTR**

**MiRNA : K12 site1**

target 5' U UCA U AGC C 3'

GGCC GU CCC UCCCCC

UUGG CG GGG AGGGGG

miRNA 3' AG UCC U U 5'

**TARGET : HMGA1-3’UTR**

**MiRNA : K12 site2**

target 5' U U CAC AUC A 3'

UC GCC G CCCUCCCCCA

AG UGG C GGGAGGGGGU

miRNA 3' U UCC GU 5'

**TARGET : HMGA1-3’UTR**

**MiRNA : K12 site3**

target 5' A UAAAGGGUGUAG CACC U 3'

UCGACCA GGGC UCCUCCCCC

AGUUGGU CCCG GGGAGGGGG

miRNA 3' U U 5'

**TARGET : IRF2BP2-3’UTR**

**MiRNA : K11**

target 5' G A UU UACCUUUUUUU UUAACUUUU A 3'

UC GAU ACAG CU AGCAUUAA

AG CUG UGUC GA UCGUAAUU

miRNA 3' C C U 5'

**TARGET : PTPN11-CDS**

**MiRNA : K11**

target 5' C U GGUCC U 3'

UAUA GGC AGCAUUA

GUGU CCG UCGUAAU

miRNA 3' AGCCU AU U 5'

**TARGET : STIP-CDS**

**MiRNA : K11**

target 5' U C UG C 3'

GACACAG CU AAGCAUUA

CUGUGUC GA UUCGUAAU

miRNA 3' AGC C U 5'

**TARGET : TPD52-3’UTR**

**MiRNA : K10**

target 5' A ACU C 3'

CUGCUU CAACACUA

GGUGAG GUUGUGAU

miRNA 3' C CCCCCCU 5'

**TARGET : TPD52-3’UTR**

**MiRNA : K4-3p**

target 5' U AUAG AA U 3'

GC AGGCC GGUAUUCUA

CG UCCGG UCAUAAGAU

miRNA 3' AGU A AG 5'

**TARGET : TP53INP1-3’UTR**

**MiRNA : K11**

target 5' U ACAUU C 3'

ACACA CUA AGCAUUAA

UGUGU GAU UCGUAAUU

miRNA 3' AGCC CC 5'

**TARGET : YWHAE-3’UTR**

**MIRNA : K11**

target 5' A U AUAG UUUUC C 3'

GGAC AC AGGCU AGCAUUA

CCUG UG UCCGA UCGUAAU

miRNA 3' AG U U 5'

**targetM** 5' A U AUAG UUUUC C 3'

GGAC AC AGGCU AcgAUaA

CCUG UG UCCGA UCGUAAU

miRNA 3' AG U U 5'

**TARGET : vIL6-3UTR**

**MIRNA : K10**

target 5' A G GU UAAAAUUUCUUAUA A 3'

GC AC GACA ACACUA

CG UG CUGU UGUGAU

miRNA 3' G AGCCCCC 5'

**targetM** 5' A G GU UAAAAUUUCUUAUu A 3'

GC AC GACA AuuuUA

CG UG CUGU UGUGAU

miRNA 3' G AGCCCCC 5'

target 5' A G GU UAAAAUUUCUUAUA A 3'

GC AC GACA ACACUA

CG UG CUGU UGUGAU

miRNA 3' G AGCCCCC 5'

**targetM** 5' A G GU UAAAAUUUCUUAUA A 3'

GC AC GACA cgcCUA

CG UG CUGU UGUGAU

miRNA 3' G AGCCCCC 5'
