# Supplementary material for: Ago HITS-CLIP Expands Understanding of Kaposi's Sarcoma-associated Herpesvirus miRNA Function in Primary Effusion Lymphomas
Source: PLoS Pathog. 2012 Aug 23;8(8):e1002884. doi: 10.1371/journal.ppat.1002884 (PMC3426530; doi:10.1371/journal.ppat.1002884)
Supplement: Table S1 — Cluster width distribution of KSHV miRNA seed match-containing clusters. (DOCX) [file ppat.1002884.s012.docx]

**Table S1: Cluster width distribution of KSHV miRNA seed match-containing clusters**

**
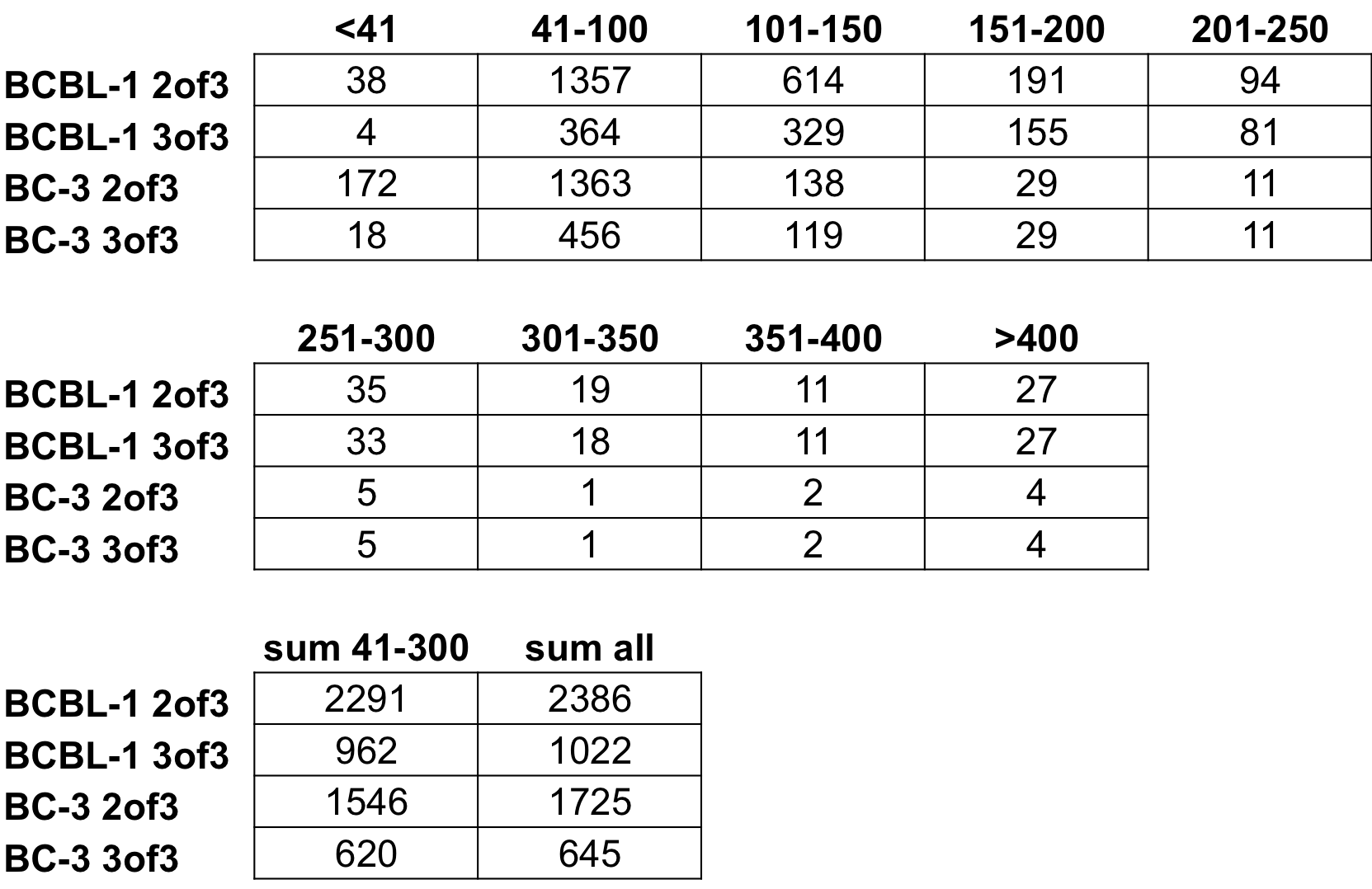
**

Based on all super clusters that align to annotated human transcripts, with coverage of at least 2 copies/cluster per 10e6 reads, and contain a KSHV miRNA seed match, at a stringency of 2of3 or 3of3. All 25 KSHV miRNAs (23 in BC-3) were included in this analysis. Clusters listed multiple times due to different miRNA seed matches in the cluster sequence were counted only once. Columns show the number of clusters of a certain width (in bp). For a graphical display see Figure 3A.
