# Supplementary material for: Ago HITS-CLIP Expands Understanding of Kaposi's Sarcoma-associated Herpesvirus miRNA Function in Primary Effusion Lymphomas
Source: PLoS Pathog. 2012 Aug 23;8(8):e1002884. doi: 10.1371/journal.ppat.1002884 (PMC3426530; doi:10.1371/journal.ppat.1002884)
Supplement: Table S2 — Target transcript abundance (A), target 3′UTR length distribution (B), and target 3′UTR GC content (C) in Ago HITS-CLIP identified miRNA targets. (DOCX) [file ppat.1002884.s013.docx]

**Table S2: Testing the possible association of the probability to identify a miRNA target with transcript frequency (A), 3’UTR length (B), and GC content (C).**

1. **Transcript frequency**

|  | **Bin 1** | **Bin 2** | **Bin 3** | **Bin 4** | **Bin 5** | **sum** |
| --- | --- | --- | --- | --- | --- | --- |
| Transcript frequency | low | low - middle | middle | middle - high | high |  |
| expected no association (%) | 20 | 20 | 20 | 20 | 20 | 100 |
| Human miRNA targets in BC-3 (%) | 2.88 | 6.69 | 13.37 | 28.09 | 48.97 | 100 |
| KSHV miRNA targets in BC-3 (%) | 4.02 | 4.55 | 11.91 | 27.98 | 51.54 | 100 |
| Human miRNA targets in BCBL-1 (%) | 4.09 | 5.72 | 16.38 | 26.85 | 46.96 | 100 |
| KSHV miRNA targets in BCBL-1 (%) | 3.61 | 5.95 | 14.56 | 24.87 | 51.01 | 100 |

1. **3’UTR length**

|  | **Bin 1** | **Bin 2** | **Bin 3** | **Bin 4** | **Bin 5** | **sum** |
| --- | --- | --- | --- | --- | --- | --- |
| Length range per bin (nt) | 1 – 289.75 | 290 – 533 | 533.5 – 863 | 863.1 – 1469 | 1469.1 – 21133.7 |  |
| Mean length per bin (nt) | 148 | 410 | 681 | 1122 | 2502 |  |
| expected no association (%) | 20 | 20 | 20 | 20 | 20 | 100 |
| Human miRNA targets in BC-3 (%) | 9.89 | 11.23 | 15.84 | 27.07 | 35.96 | 100 |
| KSHV miRNA targets in BC-3 (%) | 10.22 | 10.66 | 17.91 | 27.03 | 34.18 | 100 |
| Human miRNA targets in BCBL-1 (%) | 9.45 | 11.87 | 18.99 | 25.72 | 33.98 | 100 |
| KSHV miRNA targets in BCBL-1 (%) | 10.99 | 11.08 | 18.14 | 28.06 | 31.72 | 100 |
| expected linear association (%) | 3.04 | 8.43 | 14.00 | 23.07 | 51.45 | 100 |

1. **GC content**

|  | **Bin 1** | **Bin 2** | **Bin 3** | **Bin 4** | **Bin 5** | **sum** |
| --- | --- | --- | --- | --- | --- | --- |
| Range GC-content per bin (%) | 0 – 37.3 | 37.4 – 42.7 | 42.8 – 48.2 | 48.3 – 54.5 | 54.6 – 100 |  |
| Mean GC content per bin (%) | 33 | 40 | 45 | 51 | 60 |  |
| expected no association (%) | 20 | 20 | 20 | 20 | 20 | 100 |
| Human miRNA targets in BC-3 (%) | 33.70 | 24.90 | 15.93 | 14.75 | 10.73 | 100 |
| KSHV miRNA targets in BC-3 (%) | 30.88 | 26.70 | 15.82 | 15.16 | 11.43 | 100 |
| Human miRNA targets in BCBL-1 (%) | 31.60 | 23.84 | 16.47 | 14.99 | 13.11 | 100 |
| KSHV miRNA targets in BCBL-1 (%) | 30.21 | 23.06 | 16.00 | 15.82 | 14.92 | 100 |
| expected linear association (%) | 26.03 | 22.33 | 19.75 | 17.46 | 14.43 | 100 |

Human transcripts were grouped into 5 bins with equal number of transcripts based on their frequency, 3’UTR length, or GC content (average of all isoforms of a transcript). HITS-CLIP targets obtained for each miRNA group were associated with the bins and counted. ‘Expected no association’ and ‘expected linear association’ represent expected numbers in case of no association or a linear association, respectively between the probability to identify a target and each of the target properties. For a graphical display see Figure S3, for detailed methods see Document S1.
