# Supplementary material for: Ago HITS-CLIP Expands Understanding of Kaposi's Sarcoma-associated Herpesvirus miRNA Function in Primary Effusion Lymphomas
Source: PLoS Pathog. 2012 Aug 23;8(8):e1002884. doi: 10.1371/journal.ppat.1002884 (PMC3426530; doi:10.1371/journal.ppat.1002884)
Supplement: Text S1 — Supplementary methods and supplementary references. (DOCX) [file ppat.1002884.s020.docx]

**Ago-HITS-CLIP Expands Understanding of Kaposi’s Sarcoma-associated Herpesvirus miRNA Function in Primary Effusion Lymphomas**

Irina Haecker^1^, Lauren Gay^1^, Yajie Yang^1^, Jianhong Hu^1^, Alison M. Morse^1^, Lauren M. McIntyre^1,2^, and Rolf Renne^1,*^

^1^ Department of Molecular Genetics and Microbiology, University of Florida, Gainesville, FL, 32610, USA

^2^UF Shands Cancer Center, University of Florida, Gainesville, FL, 32610, USA

^3^UF Genetics Institute, University of Florida, Gainesville, FL, 32610, USA

**Contents list:**

- **Supplementary Methods**
- **Supplementary References**

**Supplementary Methods**

**Cell lines and growth**

BCBL-1 cells [1] and BC-3 cells [2] were cultured in RPMI 1640 medium containing 2 mM glutamine, supplemented with 10% FBS, 1 mM sodium pyruvate, and 100 U/ml penicillin and 100 µg/ml streptomycin at 37°C in 5% CO_2_ to a maximum density of 0.8x10^6^ cells/ml. HEK293 cells were cultured in Dulbecco’s Modification of Eagle’s Medium (DMEM) containing 4.5 g/L glucose and L-glutamine, supplemented with 10% FCS, 1 mM sodium pyruvate, and 100 U/ml penicillin and 100 µg/ml streptomycin at 37°C in 5% CO_2_ to maximum 90% confluence.

**Plasmids**

miRNA expression plasmids either contain a region of approximately 200 bp encompassing the pre-miRNA stem loop or the complete intronic miRNA cluster inserted into pcDNA3.1/V5/HisA and have been described by Samols et al. [3] except for miR-K12-12. For the latter, primers were designed to amplify the sequence of the pre-miRNA hairpin. The hairpin secondary structure of this sequence was confirmed in mfold. The sequence was amplified by PCR from BCBL-1 genomic DNA. Primers contained restriction sites for XhoI and HindEIII for cloning into pCDNA3.1 expression vector. The Renilla luciferase vector pRL-CMV was obtained from Promega. Firefly luciferase reporter plasmids are described elsewhere [4,5] or were created using the pGL3 promoter vector (Promega). Sequences of 3’UTRs or CDS were obtained from RefSeq. 3’UTRs were PCR amplified from BCBL-1 genomic DNA, CDS from BCBL-1 cDNA using the USB FideliTag enzyme. Primers (see TableS8) were designed such that they contained an overhang complementary to the pGL3 promoter vector sequence encompassing the XbaI and FseI restriction sites downstream of the luciferase gene. These overhangs were subsequently used to insert the PCR product into the pGL3 promoter vector by homologous recombination using the GeneArt Seamless Cloning kit (Invitrogen). The GeneArt Seamless cloning strategy for simultaneous cloning of 2 fragments was used to introduce point mutations into seed matches of selected 3’UTRs. For this, inverted, 15 bp overlapping primers were designed containing the mutated seed match sequence in the overlapping region. These were combined with the primers described above, designed for cloning the 3’UTRs into the pGL3 vector to obtain two PCR products that overlap with each other and the pGL3 vector. The corresponding pGL3 reporter-3’UTR construct served a template for these PCR reactions The two PCR products were then incubated with linearized pGL3 vector and the GeneArt enzyme mix according to the manual. In all cases obtained reporter constructs were confirmed by restriction enzyme digest and sequencing.

**Transfections and Luciferase Reporter Assays**

HEK293 cells were transfected using Mirus TransIT-293 in 24-well cell culture dishes according to the manufacturer’s protocol. Cells were seeded at a density of 1.5x10^5^ cells/well and incubated for a few hours until adherent. Each transfection reaction contained 2 ng of the pCMV-Renilla control vector, 20 ng of the Firefly pGL3 reporter construct and 0, 400 or 800 ng of the pcDNA3.1 miRNA expression vector. The different concentrations of pcDNA3.1 miRNA expression vector were complemented with 800, 400, or 0 ng of empty pcDNA3.1 vector to reach 800 ng total pcDNA3.1 in each transfection. Cells were harvested 72 hrs post transfection (72 hpt resulted in the best repression, Figure S5A) and luciferase activity was quantified using the Promega Dual Luciferase Reporter kit according to the manufacturer’s protocol. Briefly, 293 cells were lysed in 200 µl passive lysis buffer (Promega) for 30 min. 10% of each lysate was assayed for firefly luciferase activity using a FLUOstar OPTIMA reader (BMG Labtech). Firefly luciferase light units (LU) were first normalized to Renilla luciferase LU to correct for transfection efficiency, and then to the Firefly LU obtained with the empty pcDNA3.1 vector (0 ng miRNA), which were set to one (100%). Transfection assays were performed in triplicate and repeated at least 3 times. Standard deviation was calculated for triplicates and displayed as error bars in the figures. Significance of the repression of the reporter construct at the highest miRNA dose (800 ng) relative to the empty vector control (0 ng miRNA expression vector) was tested by two-tailed, unpaired t-test. Seed matches of selected targets were mutated by introducing 3-4 point mutations. These mutants were tested in an independent series of experiments, testing only 0 ng and 800 ng miRNA expression vector in combination with the unmutated or the mutated reporter construct. Significance of the reporter construct repression in presence of the miRNA compared to no miRNA, as well as derepression of the mutated compared to the unmutated reporter was tested by two-tailed, unpaired t-test.

**Western blotting and antibodies**

Immunoblotting was carried out to detect down-regulation of miRNA targets at the protein level. Cell lysates were the same as used for luciferase reporter assays and were obtained from 293 cells transfected with either 0 ng or 800 ng of KSHV miRNA. 10-12 ug of total protein per lane were separated on 10% or 12% SDS gels and transferred to PVDF membranes using standard procedures. Membranes were probed with the following antibodies: rabbit anti-TP53INP1 (eBioscience, 14-6049), rabbit anti-YWHAE (Thermo Scientific, PA5-17104)), goat anti-actin-HRP (Santa Cruz, sc-1616), and goat anti-rabbit-HRP (Jackson Immunoresearch, 111-036-047)

**Ago HITS-CLIP**

Ago HITS-CLIP procedure was performed in biological replicates as described in Chi et al. [6] with some minor modifications. Briefly, cells were harvested at a density of < 0.8x106 cells/ml, resuspended in a small volume ice cold PBS and transferred to a 10 cm cell culture dish on ice. Cells were then crosslinked once at 400 mJ/cm2 and once at 200 mJ/cm2 at 254 nm. After crosslinking cells were either stored at -80 degree as cell pellet or lysed with cell lysis buffer [7] 15 min on ice. Cell lysates were then treated with RQ1 DNase and RNase A (1:5000 – 1:10000) as described in Chi et al [6]. After pelleting of cell debris the lysates were incubated with the monoclonal anti-Ago antibody 2A8 [8], immobilized on protein G beads (Invitrogen) for 4 hrs at 4 degree. Immunoprecipitated RISC complexes were washed twice with cold high stringency buffer (15 mM Tris-HCl, pH Z5, 5 mM EDTA, 2.5 mM EGTA, 1% TX-100, 1% Na-deoxycholate, 0.1% SDS, 120 mM NaCI, 25 mM KCI), twice with high salt buffer (15 mM Tris-HCl, pH 7.5, 5 mM EDTA, 2.5 mM EGTA, 1% TX-100, 1% Na-deoxycholate, 0.1% SDS, 1 M NaCI) [9], and then as described by Chi et al. [6]. The 3’ adapter was ligated to the RNPs while still on the beads and the ligated product labeled with [γ-^32^P]-ATP. RISC complexes were then eluted from the beads and further purified on a NuPAGE gradient SDS gel (4-20%, Invitrogen) followed by transferring onto a nitrocellulose membrane (0.2 µm, Biorad) using the Invitrogen X-cell blot module. Ago-RNA complexes in a narrow region around 130 kDa were cut out from the membrane and RNA extracted and reverse transcribed. RNA extracted from 130 kDa gave rise to two major DNA species after PCR amplification, a shorter, very pronounced band migrating at 90 bp after the first PCR (15 cycles), and at 110 bp after 2^nd^ PCR (10-12 cycles), further on treated as miRNA library, and a longer product migrating more diffusely at 80-110 bp and 140-160 bp, respectively (mRNA library). Both DNA species were extracted from the gel and sequenced in 40 bp runs on an Illumina GAIIx sequencer. Ago-CLIP was performed in 3 biological replicates (BR) for each cell line. For RNA adapter and primer sequences see Chi et al. [6].

**Bioinformatics**

**Adapter removal, sequence annotation and data visualization**

After adapter trimming, reads were aligned to the hg19 version of the UCSC human genome assembly and to the complete KSHV genome (NCBI accession number nc_009333) using Bowtie [10]. For visual inspection of the libraries, aligned sequence files were converted into pileup format and then to wiggle files for upload to the UCSC Genome Browser (<http://genome.ucsc.edu/>) as custom track.

**miRNA library analysis**

Sequencing reads obtained from the miRNA libraries were analyzed for their miRNA content using an in-house algorithm: sequence tags were aligned to miRBase (<http://www.mirbase.org/>; release 17) using BLAST; all 19-24 nt long sequence tags aligning to miRBase human and KSHV mature miRNA sequences with at most 1 mismatch were counted as hit. Results were confirmed with the miRDeep2 software package (<http://www.mdc-berlin.de/de/research/research_teams/systems_biology_of_gene_regulatory_elements/projects/miRDeep/index.html>) [11]. The sequences of all mature and precursor miRNAs and were obtained from miRBase. In the miRDeep analysis, reads were first collapsed to the reference of human genome and KSHV genome. The reads that were shorter than 17 bases after adapter removal were discarded. The collapsed reads were then compared with the sequences of known miRNAs. Reads mapped to the mature, loop and star sequences were identified according to the miRDeep2 algorithm

MiRNAs were then ranked based on their read frequency in the Ago-miRNA libraries. For this the miRNA counts of the biological replicates were averaged for each miRNA. A miRNA was only considered as being present in the RISC-complexes of each cell line if the average counts over BRs were ≥ 10. For comparison of different libraries/sequencing runs the miRNA frequencies were normalized to the total sequencing reads of the sample and rescaled to a standard sample size of 1x10^6^ sequencing reads. For miRNA target search the sequencing reads obtained for human miRNAs belonging to the same miRNA family and having the same seed sequence (nt 2-8) were summarized and displayed under the miRNA family name. The miRNA rankings were adjusted accordingly and the top 30 human miRNAs/miRNA families were selected for target search.

**mRNA library read annotation and cluster formation for target search**

For both cell lines the mRNA libraries of 3 BRs were sequenced. Moreover, for the first BR of BCBL-1, two technical replicates (TR) were sequenced. Sequencing reads (.fastq) were uploaded to the CLIPZ database, an open source software package specifically developed for the analysis of HITS-CLIP and PAR-CLIP data ([www.clipz.unibas.ch](http://www.clipz.unibas.ch); [12], and annotated to the human genome. Clusters of overlapping reads were then identified using CLIPZ’s sequence cluster function. Settings chosen for cluster formation were ‘clusters containing mRNA’ and ‘clusters built on genome’. Next, BRs were analyzed for the presence of overlapping clusters (super clusters), i.e. if the clusters found in BR1 overlap with clusters in BR2 and/or BR3 and vice versa, using the CLIP super cluster tool. Settings chosen for super cluster formation were ‘clusters containing mRNA’ and ‘clusters built on genome’. The cluster overlap had to be at least one nucleotide (distance set to ‘zero’) to form a super cluster. For clusters of BCBL-1 BR1 to be considered in this analysis they had to be present (i.e. overlapping) in both TRs. Super cluster search was performed at 3 different stringencies: i) all clusters and super clusters present in one or more BRs (‘1of3’; these super clusters were not considered for HITS-CLIP target search; they were needed for other analyses (see below and comparison with published target lists)), ii) super clusters present in at least 2 of 3 BRs (‘2of3’), and iii) super clusters present in all 3 BRs (‘3of3’). CLIPZ provides an annotation summary tool for raw reads, but not for read clusters. To obtain an annotation summary for clusters/super clusters of reads, we used the 1of3 super cluster files and extracted the sequences of all clusters that were 41-200 nt long (due to the read length of 40 nt every cluster with a width of <41 nt must originate from breakdown products and is therefore not considered as valid target cluster; 200 nt is the max. sequence length that can be annotated by the current version of CLIPZ). These were uploaded to the CLIPZ database as .fa file, annotated to the human genome and analyzed with the Summaries tool.

**miRNA target search**

Super clusters obtained at different analysis stringencies, as described above, were initially searched for 7-mer seed matches (nt 2-8) of all 25 KSHV and the top 30 human miRNAs (or miRNA families) using the CLIPZ reverse complement search tool. Seed matches were searched in clusters called at all analysis stringencies (1of3, 2of3, 3of3), but for the creation of the Ago-HITS-CLIP miRNA target lists only super clusters identified at the stringencies 2of3 and 3of3 were considered. For KSHV miRNA target analysis miR-K12-1*, miR-K12-2*, miR-K12-5*, miR-K12-10a*, miR-K12-11*, and miR-K12-6-5p were excluded because they were recovered from RISC complexes at very low frequencies. (In BC-3 we additionally excluded miR-K12-9 and miR-K12-9* because they are not expressed). It is likely that clusters with seed matches for these miRNAs were not recovered due to a true, biologically important interaction with any of these miRNAs, but rather due to other targeting interactions (e.g. targeting by another miRNA). For miR-K12-6-5p targets for example, we observed a strong overlap (> 60%) with targets of the much more frequent human miR-15 due to a 6-mer seed sequence overlap. The cut-off for the exclusion of these miRNAs was selected because across cell lines we consistently observed a marked drop in read frequency between the first excluded miRNA (miR-K12-6-5p) and last included miRNA (K12-12* in BCBL-1 and K12-6-3p in BC-3).

The seed match containing clusters were further filtered excluding first all clusters aligning to non-annotated regions of the genome, then clusters with low coverage (< 2 copies/cluster/10e6 reads), and finally clusters < 41 nt (due to the sequencing read length of 40 nts) and > 300 nt wide. All cluster wider than 300 bp were saved in separate lists. Visual inspection of wider clusters often showed overlapping peaks with multiple miRNA target sites. These larger clusters were excluded from the above KSHV miRNA target tables, as it was not possible to determine without individual visual inspection which of the target sites represented the most likely targeting interaction.

**Investigation of bias for transcript frequency, 3’UTR length and GC content in putative miRNA targets**

The probability to identify a transcript as target of a miRNA can be influenced by several factors, e.g. transcript frequency, 3’UTR length and GC content: theoretically the probability to capture a transcript increases with its expression level and its length (the longer the 3’UTR the more potential miRNA target sites it may contain), and decreases with the GC content (highly structured 3’UTRs are potentially less accessible for RISC complexes).

We first tested for a bias due to transcript abundance. For this the expression profiles for BC-3 and BCBL-1 published by [13] were downloaded. Each cell line had two replicates of arrays. The signal for each probe set was determined as the average from the two replicates. For genes with multiple probe sets, the expression level was determined as the average signal of all corresponding probe sets. Then the profiles were annotated with gene symbols. Next, the gene-level signals were categorized into five bins with equal number of genes: low, low-middle, middle, middle-high and high. Then the target genes were combined with the expression signals, and the number of target genes in each bin of expression level was counted. This was done separately for human and KSHV miRNA targets in both cell lines. The association between the expression level and target identification was measured using Chi-square test. The test was significant for every set of miRNA targets, indicating association between expression level and target identification. The trend of the association across expression levels was measured using Cochran-Mantel-Haenszel (CMH) test, which showed significant results that the associations were different across the levels of expression. Overall, we detected a bias for more abundant transcripts (Table S2A and Figure S3A), which is expected since more frequent transcripts will be more frequently targeted and therefore also be co-immunoprecipitated more often.

We then tested for bias due to 3’UTR length. The length was obtained for every human transcript using a Perl script. For each 3’UTR it was determined as the average of all the isoforms of a transcript (computing the longest isoform instead of the average slightly changed the numbers but not the overall trend). Similar to the transcript abundance analysis, the 3’UTR length was categorized into five equally sized bins and compared with the targets. To project the expected target numbers in case of a linear association between 3’UTR length and probability to identify a target, the average 3’UTR length (mean) of all transcripts in each bin and the fold difference between average lengths of adjacent bins was calculated. From this the expected distribution of transcripts across bins in case of linear association was deducted. A similar analysis was conducted for GC content of 3’UTRs. Chi-square tests showed association between 3’UTR length or GC content and target identification. CMH test was significant for all combinations. Overall we detected a slight but significant bias for longer 3’UTRs, which was much lower than expected from a linear association between UTR length and probability of target identification (Table S2B and Figure S3B) For example, although the average 3’UTR length in the bin with the longest 3’UTRs was 16-fold longer than the average in the shortest bin, we found only 3-fold more targets in the bin with the longest 3’UTRs vs the shortest. For the GC content the analysis identified enrichment for targets with low GC content that was slightly stronger than expected from a linear association (Figure S3C and Table S2C). 3’UTRs with lower GC content are probably less structured and therefore better accessible to RISC complexes.

**Correction for potential 5’UTR length bias**

5’UTRs on average are much shorter (~ 6-fold) than 3’UTRs. Accordingly, the probability to contain a seed match site is lower. In our analysis 4% of HITS-CLIP clusters align to 5’UTR sequences, which is significantly lower than approx. 30% aligning to 3’UTRs. Assuming that the probability to detect clusters on 5’UTRs increases linearly with the length then at the same average length we would expect almost as many clusters aligning to 5’UTRs as to 3’UTRs. However, we have shown above that the probability to identify a target is not linearly correlated with the 3’UTR length. Instead of a 16-fold increase of target numbers between the shortest and longest 3’UTR bin as expected for a linear correlation we observe only a 3.36-fold increase (average over all groups, range 2.9-3.6-fold), i.e. only 20% of what would be expected from a linear correlation. Applying these results to the 5’UTR would result in a 1.2-fold adjustment (not 6-fold) of interaction sites mapping to 5’UTRs. When comparing bin 1 and bin 3 (Figure S3B, Table S2B) which roughly correspond to the average 5’UTR and 3’UTR lengths, respectively, we observe a less than 2-fold increase in target numbers with increased 3’UTR length. Thus, even with these adjustments our findings are still highly relevant.

**Analysis of the KSHV miRNA target overlap between cell lines**

KSHV miRNA target lists of analysis stringencies 2of3 and 3of3, created as described above, were compared for targets found in BCBL-1 and BC-3. The analysis excluded targets of miR-K12-1*, miR-K12-2*, miR-K12-5*, miR-K12-10a*, miR-K12-11*, and miR-K12-6-5p in both cell lines. In addition, miR-K12-9 and miR-K12-9* were excluded in BC-3 but not in BCBL-1, contributing to the differential set of targets between the two cell lines. Venn diagrams were created using the Venn Diagram Plotter v1.4.3740 from Pacific Northwest National Laboratory (<http://omics.pnl.gov/software/VennDiagramPlotter.php>).

**Comparison of targets identified by Ago-HITS-CLIP with published KSHV miRNA targets**

For comparison of the KSHV miRNA targets identified by Ago-HITS-CLIP with previously published targets, our target lists were adjusted such that they represent the closest possible match to the conditions used in the published lists. i.e. for comparison with the BCBL-1 and DG75 target lists from Dolken et al. [14] (2 biological replicates) the stringency 2of3 was used and all KSHV miRNAs were included (except for miR-K12-9 and -9*in BC-3). For comparison with the KSHV miRNA targets identified by Gottwein et al. [15] in BC-3 (1 biological replicate) we used the BCBL-1 and BC-3 target lists created at the analysis stringency 1of3 and included all KSHV miRNAs except for miR-K12-5*, -9 and -9*. Using these criteria HITS-CLIP recovered 42% of the putative targets identified by PAR-CLIP in BC-3. In a second, more stringent comparison, we sorted out all putative PAR-CLIP target clusters that were recovered due to a 7mer1A seed match, and moreover, all clusters containing a miR-K12-10a_+1_5 7mer1A or 8mer1A seed match, as these were not part of our analysis and do not include the complete miR-K12-10a 7mer2-8 seed match. Using these criteria HITS-CLIP recovered 47% of the putative targets identified by PAR-CLIP in BC-3.

**Gene Ontology analysis**

Gene Ontology (GO) analysis was performed using the web-accessible database DAVID (<http://david.abcc.ncifcrf.gov>; [16,17]. DAVID annotation stringency was set to ‘High’. Only the GO FAT terms for biological processes (GOTERM_BP_FAT) were included in the analysis. GO analysis was performed on KSHV miRNA target lists of stringency 3of3, i) for all targets found in a cell line, ii) for the cell line-specific targets, and iii) for common targets between the two cell lines. Target gene enrichments were calculated against the background of the complete human transcriptome and against the background of the cell line-specific transcriptomes [13]. For the latter, we selected all genes that were reported as present ‘P’ or moderate ‘M’ in both replicates of the microarray data sets. For the common targets the combined BCBL-1 and BC-3 transcriptomes were used as cell line-specific background. Only pathways significantly enriched against the cell line-specific background (p ≤ 0.05) or against both backgrounds (p ≤ 0.1 against cell line-specific transcriptome and p ≤ 0.01 against total human transcriptome) were considered.

**Supplementary References**

1. Renne R, Zhong W, Herndier B, McGrath M, Abbey N, et al. (1996) Lytic growth of Kaposi's sarcoma-associated herpesvirus (human herpesvirus 8) in culture. Nat Med 2: 342-346.

2. Arvanitakis L, Mesri EA, Nador RG, Said JW, Asch AS, et al. (1996) Establishment and characterization of a primary effusion (body cavity-based) lymphoma cell line (BC-3) harboring kaposi's sarcoma-associated herpesvirus (KSHV/HHV-8) in the absence of Epstein-Barr virus. Blood 88: 2648-2654.

3. Samols MA, Hu, J., Skalsky, R.L., Maldonado, A.M., Riva, A., Lopez, M.C., Baker, H.V., and R. Renne (2007) Identification of cellular genes targeted by KSHV-encoded microRNAs. PLoS Pathog.

4. Skalsky RL, Samols MA, Plaisance KB, Boss IW, Riva A, et al. (2007) Kaposi's sarcoma-associated herpesvirus encodes an ortholog of miR-155. J Virol 81: 12836-12845.

5. Boss IW, Nadeau PE, Abbott JR, Yang Y, Mergia A, et al. (2011) A KSHV encoded ortholog of miR-155 induces human splenic B-cell expansion in NOD/LtSz-scid IL2R{gamma}null mice. J Virol.

6. Chi SW, Zang JB, Mele A, Darnell RB (2009) Argonaute HITS-CLIP decodes microRNA-mRNA interaction maps. Nature 460: 479-486.

7. Hafner M, Landthaler M, Burger L, Khorshid M, Hausser J, et al. (2010) Transcriptome-wide identification of RNA-binding protein and microRNA target sites by PAR-CLIP. Cell 141: 129-141.

8. Nelson PT, De Planell-Saguer M, Lamprinaki S, Kiriakidou M, Zhang P, et al. (2007) A novel monoclonal antibody against human Argonaute proteins reveals unexpected characteristics of miRNAs in human blood cells. RNA 13: 1787-1792.

9. Hinck L, Nathke IS, Papkoff J, Nelson WJ (1994) Dynamics of cadherin/catenin complex formation: novel protein interactions and pathways of complex assembly. J Cell Biol 125: 1327-1340.

10. Langmead B, Trapnell C, Pop M, Salzberg SL (2009) Ultrafast and memory-efficient alignment of short DNA sequences to the human genome. Genome Biol 10: R25.

11. Friedlander MR, Chen W, Adamidi C, Maaskola J, Einspanier R, et al. (2008) Discovering microRNAs from deep sequencing data using miRDeep. Nat Biotechnol 26: 407-415.

12. Khorshid M, Rodak C, Zavolan M (2011) CLIPZ: a database and analysis environment for experimentally determined binding sites of RNA-binding proteins. Nucleic Acids Res 39: D245-252.

13. Fan W, Bubman D, Chadburn A, Harrington WJ, Jr., Cesarman E, et al. (2005) Distinct subsets of primary effusion lymphoma can be identified based on their cellular gene expression profile and viral association. J Virol 79: 1244-1251.

14. Dolken L, Malterer G, Erhard F, Kothe S, Friedel CC, et al. (2010) Systematic analysis of viral and cellular microRNA targets in cells latently infected with human gamma-herpesviruses by RISC immunoprecipitation assay. Cell Host Microbe 7: 324-334.

15. Gottwein E, Corcoran DL, Mukherjee N, Skalsky RL, Hafner M, et al. (2011) Viral microRNA targetome of KSHV-infected primary effusion lymphoma cell lines. Cell Host Microbe 10: 515-526.

16. Huang da W, Sherman BT, Lempicki RA (2009) Systematic and integrative analysis of large gene lists using DAVID bioinformatics resources. Nat Protoc 4: 44-57.

17. Huang da W, Sherman BT, Lempicki RA (2009) Bioinformatics enrichment tools: paths toward the comprehensive functional analysis of large gene lists. Nucleic Acids Res 37: 1-13.
